# Creating alternative seafood flavour from non-animal ingredients: A review of key flavour molecules relevant to seafood

**DOI:** 10.1016/j.fochx.2024.101400

**Published:** 2024-04-24

**Authors:** Jiaqiang Luo, Damian Frank, Jayashree Arcot

**Affiliations:** a Food and Health, School of Chemical Engineering, Faculty of Engineering, UNSW Sydney, Kensington, NSW 2052, Australia; b All G Foods, Waterloo, NSW 2017, Australia

**Keywords:** Macronutrient, Odourants, Olfactometry, Plant-based, Volatiles

## Abstract

This review summarises current knowledge of the molecular basis for flavour profiles of popular seafood types (crustacean (crab, lobster, prawn, etc.), mollusc (oyster, squid, etc.), oily fish (salmon, sardine, etc.) and white fish (barramundi, turbot, etc.)), and provides a foundation for formulating improved plant-based seafood alternative (PBSA) flavours. Key odour-active volatile molecules were identified from a systematic review of published olfactometry studies and taste-active compounds and macronutrient profiles of different seafood species and commercial PBSAs from nutrition databases were compared. Ingredients commonly used in commercial BPSAs and new potential sources of flavouring agents are evaluated. While significant challenges in replicating seafood flavour and texture remain, this review provides some insights into how plant-based ingredients could be applied to improve the acceptability of PBSAs.

## Highlights

• Odour profiles for seafood species were built from published olfactometry data.
• Non-volatile data were obtained from food composition databases.
• Differences were found between seafood species based on odour impact volatiles.
• Raw seafood has more odour differences than cooked.
• Plant-based and authentic seafood differ in no-volatile profiles.

## 1 Introduction

World population growth and increasing incomes are driving an unsustainable demand for seafood while, alarmingly, over 90% of wild fisheries are classified as overfished or harvested at maximum capacity (FAO, 2020). Climate and other changes to marine environments are already affecting seafood yields and their nutritional and sensory profiles (Shalders, Champion, Coleman, & Benkendorff, 2022). The global food protein landscape is undergoing a significant transformation, with an emphasis on more sustainable and healthier alternatives to traditional animal-based protein products. Plant-based alternatives to terrestrial animal protein (red meat), replicating the taste, texture, and nutritional profiles of using plant-derived ingredients, are already commonplace in many countries. Forecasts predict the market for alternative protein to increase to US $85 billion by the year 2030 (Abbaspour, Sanchez-Sabate, & Sabaté, 2023). PBSAs are a promising part of this growing protein segment, although their development and commercialisation are less advanced compared to red meat analogues (Alcorta, Porta, Tarrega, Dolores Alvarez, & Pilar Vaquero, 2021).

The concept of PBSAs can trace its origins to ancient culinary practices, wherein ingredients such as tofu, tempeh and konjac (glucomannan) and additional flavourings have been utilised, to emulate the textures and flavour profiles of seafood with varying degrees of success (Alcorta et al., 2021). More recently, advances in food science and technology have enabled the development of products that more closely resemble the sensory and nutritional attributes of authentic seafood (Houicher, Bensid, Regenstein, & Özogul, 2021).

As with other alternative protein categories, a primary barrier to greater consumer acceptance lies in the often-disappointing sensory characteristics (texture and flavour) of these products (Kim, Caputo, & Kilders, 2023). The complex and subtle flavour profile of freshly cooked seafood is a result of a combination of volatile (odour, aroma) and non-volatile (taste) chemical inputs, perceived through olfactory receptors in the nasal epithelium and taste receptors in the oral cavity, respectively.

Much of the unique flavours of fresh seafood are directly incorporated from the marine biota – for example, omega-3 fatty acids and brominated compounds from seaweed present in the sea creature's diet. Others are the direct consequence of molecules essential for the cellular survival in a saline and aquatic environment, including osmolytes, molecules involved in protecting cells from changes in water pressure, examples being trimethylamine oxide (TMAO), betaine (*N*,*N*,*N*-trimethyl glycine), proline, taurine and others (Yancey, Blake, & Conley, 2002). Apart from their physiological role, many of these molecules add to the unique flavour and nutritional proposition of seafood. The enzymatic and bacterial degradation of TMAO to form the highly pungent volatile compound trimethylamine (TMA), is strongly associated with fishy odour and seafood spoilage, for example.

Additional odour-impact volatiles in raw seafood are formed via the oxidation of polyunsaturated fatty acids and the degradation of sulphur amino acids (Jones et al., 2022). A combination of these and other odour-active volatile molecules underpin the familiar and delicate oceanic odours found in raw seafood. While fresh raw seafood typically possesses a mild, clean ocean flavour, thermal processing methods like steaming, grilling and roasting induce a range of complex chemical transformations. During cooking, heat promotes the hydrolysis and oxidation of lipids, as well as the breakdown of nitrogen-containing compounds, including proteins and amino acids (Nieva-Echevarría, Manzanos, Goicoechea, & Guillén, 2017). Oily fish are renowned for their high content of omega-3 polyunsaturated fatty acids which easily breakdown to form an array of odour-active aldehydes and alcohols like (*E*,*E*)2,4-heptadienal, (*E*)-2-hexanal, (*E*,*E*,*E*)-2,4,7-decatrienal, (*E*)-2-nonenal, 1-octen-3-ol and 1,5-octadien-3-ol, imparting ‘fried fish’, ‘typical fresh’, ‘marine-like’, or ‘fishy’ odours (Hammer & Schieberle, 2013; Nogueira, Scolaro, Milne, & Castro, 2019). Furthermore, interactions between dicarbonyl compounds (e.g. sugar and lipid breakdown intermediates) and amino acids through thermally induced Strecker and Maillard reactions, form a diverse range of odour-active volatiles such as, methional, 3-methylbutanal, 2,3-pentanedione, 2,3-butanedione and alkyl-pyrazine compounds (Xu et al., 2013; Yaylayan & Keyhani, 1999). Differences in the initial concentration of non-volatile precursors may influence the odour potential of heated seafood samples.

The distinctive flavour profiles of seafood are also shaped by the presence of non-volatile taste compounds. Most importantly, the umami taste related compounds, free glutamic acid and 5′-nucleotides like inosine monophosphate (IMP), guanosine monophosphate (GMP), are well-known contributors to seafood taste (Sarower, Hasanuzzaman, Biswas, & Abe, 2012). Other free amino acids like aspartic, glycine, alanine, arginine, proline, valine, methionine, phenylalanine, and tyrosine, may also play a role in seafood flavour. Organic acids, like lactic and succinic acid acids may modulate seafood flavour perception (Shi, Pu, Zhou, & Zhang, 2022). Furthermore, sodium and potassium are minerals of obvious importance to seafood salty flavour (Chen & Zhang, 2007; Rotzoll, Dunkel, & Hofmann, 2006). Sodium and potassium cations in combination with glutamate, phenylalanine and tyrosine synergistically enhance umami taste - even below individual threshold levels (Lioe, Apriyantono, Takara, Wada, & Yasuda, 2005). Notably, these umami compounds provide fundamental characteristics of seafood taste (Sarower et al., 2012). Glycine and alanine are noted for imparting a sweet taste, while arginine and other hydrophobic amino acids can yield an unpleasant bitter taste at high concentrations (Shallenberger, 1993).

There is a noticeable gap in capturing more authentic and desirable flavour signatures of seafood alternatives. While several literature reviews on PBSA have been published (Kazir & Livney, 2021; Nowacka et al., 2023), none have specifically focused on the molecules underpinning seafood flavour. In this review, our primary objective was to obtain a clearer understanding of the (bio-) chemical basis for seafood flavour in general, and also to elucidate those responsible for the distinctive flavour attributes of specific seafood species, based on published research, for example through a systematic review of published olfactometry studies. We also compared the non-volatile composition of different seafood species from nutrient databases and commercial ingredient labels with the aim to understand potential variations in taste-active compounds and odour precursors. Commonly used flavouring agents used in the formulation of commercial PBSAs are evaluated and future prospects discussed.

## 2 Method

### 2.1 Systematic literature search for olfactometry data

Gas chromatography–olfactometry (GC–O) is a technique designed to identify odour-active volatiles within a larger array of non-odour active volatiles typically present in chromatographic outputs (Dunkel et al., 2014). Although hundreds of volatiles have been identified across seafood species by gas chromatography–mass spectrometry (GC–MS), only a small subset has been confirmed by GC-O to actually make a contribution to seafood flavour (Jones et al., 2022). Hence, we conducted a systematic review of published GC-O studies to confirm the key odour-active volatile compounds identified in seafood, using a search strategy similar to Jones et al. (2022). Databases including Scopus, Web of Science, ProQuest Science & Technology, and Scifinder-n were searched in August 2023 utilising the following key words: “seafood*” OR “bass” OR “barramundi” OR “clam” OR “cod” OR “crab” OR “hake” OR “lobster” OR “mullet” OR “mussel” OR “oyster” OR “prawn” OR “shrimp” OR “salmon” OR “sardine” OR “scallop” OR “squid” OR “trout” OR “tuna” AND “GC–O" OR “gas chromatography olfactometry”. The selection of key words was based on the most common seafood varieties available in Australian and other markets. Studies employing deep-frying, seasoning, or smoking treatments were excluded, as these processes introduce volatile compounds that do not originate from the seafood.

In contrast to Jones et al. (2022) our study strictly adheres to seafood (saltwater) and not freshwater species. Most commercially available PBSAs are designed to mimic popular saltwater species such as salmon, tuna and prawn (**Supplementary file 1**). Hence, this review is restricted to saltwater species with the focus on crustacean (e.g., prawn, crab, lobster etc.), molluscs (oyster, clam, mussel etc.) and finfish. According to their omega-3 fatty acid content, finfish species were classified into “oily” fish (> 500 mg/100 g, e.g., salmon, trout, yellowtail, etc.) and white fish (< 500 mg/100 g, barramundi, mullet, turbot etc.). Note that depending on the diet and rearing method, the final omega-3 lipid content can vary significantly within different species.

### 2.2 Integration of GC–O data from selected studies

Quantitative GC–O results from the selected published studies were expressed using either direct perceived intensity, frequency of detection or flavour dilution (FD) factor. Intensity and frequency of detection data were directly rescaled to a range of 0–100. FD data were first subjected to a Box-Cox transformation before being rescaled to 0–100 scores. The transformation is particularly useful when comparing FD value data with intensity and frequency of detection data As FD values can vary from 1 to over 2000 and datasets often have means greater than their medians, this approach can help in transforming skewed data, rendering it closer to a normal distribution (Sakia, 1992).

To avoid the inclusion of spurious volatiles, only compounds found in multiple studies for each species were included. The relative odour activity (ROA) for each volatile compound was calculated by multiplying its group mean value by the ratio of frequency of presence to the group size.

### 2.3 Macronutrient and micronutrient composition of seafood and PBSAs

Seafood nutrient data were obtained from databases of the U.S. Department of Agriculture Agricultural Research Service and Food Standards Australia New Zealand, accessible at https://fdc.nal.usda.gov and https://www.foodstandards.gov.au, respectively. Further information for 41 commercial PBSA products sourced from North America (*n* = 21), Asia (*n* = 15), Europe (*n* = 4) and Brazil (*n* = 1) was obtained from the nutritional fact labels. These detailed data as well as ingredient lists of PBSAs are provided in **Supplementary material 1 & 2**.

### 2.4 Statistical analysis

Group means of total fat, saturated fat, protein, carbohydrate, sugar and dietary fibre were compared by one-way ANOVA followed by Tukey's post-hoc test using XLSTAT software version: 2023.2.0 (1411) (Lumivero, Denver, CO). Histograms were generated using GraphPad Prism version: 9.5.1 (733) (GraphPad Software, Boston, MA). Principal component analyses (PCA) were performed on raw and cooked seafood GC–O data based on the top 15 odour impact compounds with the highest ROAs for crustaceans, molluscs, white fish and oily fish. PCA was also conducted for non-volatile compounds and PCA biplots were visualised using XLSTAT software. For a better visualisation of the contribution of odour-active volatiles to differences between seafood species groups, those with the most significant correlations with either PC1 or PC2 were retained in the biplots.

## 3 Results and discussions

### 3.1 Key odour-active compounds in seafood

**Fig. 1:**
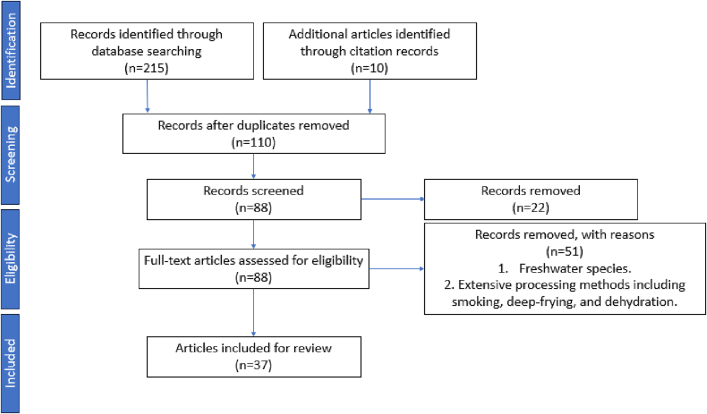
PRISMA diagram illustrating the selection process for studies on aroma active compounds in seafood samples.

Data from 37 quantitative GC–O studies were selected for the final statistical analysis (Table 1). The protocol of this workflow is illustrated in Fig. 1. Eight studies were found for crustaceans (*n* = 1 raw and cooked, *n* = 7 cooked), 8 for molluscs (*n* = 3 raw, *n* = 5 cooked), 14 for oily fish (9 raw, 5 cooked) and 8 for white fish (n = 1 raw and cooked, n = 3 raw, *n* = 4 cooked). The majority of the studies used boiling, blanching or steaming (*n* = 15) for cooking. It should be noted, the cooking method has a considerable impact on the type and amount of volatiles formed, for example steaming or boiling compared to grilling or baking. Results of identified odourants in seafood and their corresponding odour qualities from both qualitative and quantitative olfactometry studies are summarised in **Supplementary Tables S1–3**.

**Table 1:** Selection of studies included in the multivariate analysis.

	**Sample** **condition**	**Cooking temperature, and time**	**Wild-caught/aquacultured**	**Extraction method**	**Capillary column**	**Data type**	**Number of unique samples**	**Total number OA volatile identified**	**Reference**
***Crustacea***									
*Crab*									
Blue crab (*Collinectes sopidus*)	Boiled	Not mentioned	Not mentioned	SDE	Supelcowax 10	FD factor	1	7	(Chung & Cadwallader, 1994)
Blue crab (*Collinectes sopidus*)	Boiled	Not mentioned	Not mentioned	SDE	Supelcowax 10	FD factor	1	5	(Chung, Chen, & Cadwallader, 1995)
Mangrove crab (*Scylla serrata*)	Steamed	20 min	Not mentioned	SDE	DB-5MS	FD factor	1	26	(Yu & Chen, 2010)
*Lobster*									
American lobster (*Homarus americanus*)	Steamed	Until 80 °C central temperature	Not mentioned	SDE	DB-Wax, DB-5MS	FD factor	1	23	(Lee, Suriyaphan, & Cadwallader, 2001)
Norway lobster (*Nephrops norvegicus*) American lobster (*Homarus americanus*)	Blanched	100 °C, 2 min (Norway lobster); 4 min (American lobster)	Wild-caught	SDE	DB-FFAP, DB-5	FD factor	2	26	(Mall & Schieberle, 2016)
*Prawn*									
Prawn (*Litopenaeus vannamei*)	Raw, blanched	100 °C, 1 min	Aquacultured	SDE	DB-FFAP, DB-5	FD factor	2	38	(Mall & Schieberle, 2016)
Red shrimp (*Pleoticus muelleri*)	Baked	100 °C, Until 70 °C central temperature	Wild-caught	SAFE	TC-Wax	FD factor	1	17	(Okabe et al., 2019)
***Mollusca***									
*Clam*									
Clam (*Meretrix lusoria*)	Boiled	30 min	Wild-caught	SAFE	OV-1	FD factor	1	11	(Sekiwa, Kubota, & Kobayashi, 1997)
Clam (*Ruditapes philippinarum*)	Boiled	3 min	Wild-caught	SAFE	HP-5MS	Direct intensity	1	18	(Huang et al., 2021)
*Mussel*									
Mussel (*Mytilus edulis*)	Steamed	20 min	Aquacultured	SDE	DB-Wax	Direct intensity	1	16	(Le Guen, Prost, & Demaimay, 2000)
Mussel (*Mytilus edulis*)	Steamed	20 min	Aquacultured, wild-caught	SDE	DB-Wax	Detection frequency	2	20	(Le Guen, Prost, & Demaimay, 2001)
*Oyster*									
Oyster (*Crassostrea gigas*)	Raw	–	Aquacultured	P&T (sample homogenate)	DB-Wax	Detection frequency	1	12	(Piveteau et al., 2000)
Oyster (*Crassostrea gigas*)	Raw	–	Aquacultured	SDE	DB-Wax	Direct intensity	1	17	(Pennarun et al., 2002)
Oyster (*Crassostrea gigas*)	Raw	–	Aquacultured	SDE	DB-Wax	Direct intensity	4	27	(Pennarun et al., 2003)
*Squid*									
Squid (*Illex argentinus*)	Boiled	30 min	Not mentioned	HS-SPE (sample homogenate)	DB-Wax	Direct intensity	1	10	(Carrascon, Escudero, Ferreira, & Lopez, 2014)
***Oily fish***									
*Salmon*									
Atlantic salmon (*Salmo salar*)	Boiled	15 min	Not mentioned	P&T (sample homogenate)	RTX-5	FD factor	1	15	(Milo & Grosch, 1996)
Atlantic salmon (*Salmo salar*)	Raw	–	Not mentioned	SDE	DB-5MS	Direct intensity	1	58	(Varlet et al., 2006)
Atlantic salmon (*Salmo salar*)	Raw	–	Not mentioned	SAFE	DB-Wax	FD factor	1	31	(Guo et al., 2021)
*Sardine*									
Moroccan sardine (*Sardina pilchardus*)	Raw	–	Wild-caught	SAFE	DB-5	FD factor	1	10	(Triqui & Bouchriti, 2003)
Moroccan sardine (*Sardina pilchardus*)	Raw	–	Wild-caught	P&T (sample homogenate)	DB-Wax	Direct intensity	1	22	(Prost, Hallier, Cardinal, Serot, & Courcoux, 2004)
Moroccan sardine (*Sardina pilchardus*)	Raw	–	Wild-caught	HS-SPME (sample homogenate)	DB-Wax	Direct intensity	1	15	(Ganeko et al., 2008)
*Trout*									
Trout (*Salmo fario*)	Boiled	10 min	Not mentioned	P&T (sample homogenate)	RTX-5	Lowest perceivable volume	1	12	(Milo & Grosch, 1995)
Brown trout (*Salmo trutta*)	Raw	–	Aquacultured	SDE	BPX-70	Detection frequency	3	23	(Sérot et al., 2002)
Rainbow trout (*Oncorhynchus mykiss*)	Thermal immersed	80 °C, 1 min	Not mentioned	SDE	DB-5	Direct intensity	1	31	(Selli et al., 2006)
Rainbow trout (*Oncorhynchus mykiss*)	Raw	–	Aquacultured	SAFE	DB-5	FD factor	1	74	(Mahmoud & Buettner, 2017)
Rainbow trout (*Oncorhynchus mykiss*)	Raw	–	Aquacultured	SAFE	DB-Wax	FD factor	8	16	(Cengiz et al., 2023)
*Yellowtail*									
Yellowtail (*Seriola quinqueradiata*)	Raw	–	Not mentioned	HS-SPME (sample homogenate)	DB-Wax	FD factor	1	12	(Tanimoto et al., 2018)
Yellowtail (*Seriola quinqueradiata*)	Raw	–	Not mentioned	HS-SPME (sample homogenate)	DB-Wax	Direct intensity	1	10	(Kitabayashi et al., 2019)
Yellowtail (*Seriola quinqueradiata*)	Thermal immersed	90 °C, 6 min; 80 °C, 4 min; 63 °C, 30 min	Not mentioned	HS-SPME (sample homogenate)	DB-Wax	Direct intensity	3	10	(Hamakawa et al., 2021)
***White fish***									
*Cod*									
Atlantic cod (*Gadus morhua*)	Boiled	10 min	Not mentioned	P&T (sample homogenate)	RTX-5	Lowest perceivable volume	1	10	(Milo & Grosch, 1995)
Atlantic cod (*Gadus morhua*)	Boiled	15 min	Not mentioned	P&T (sample homogenate)	RTX-5	FD factor	1	14	(Milo & Grosch, 1996)
Atlantic cod (*Gadus morhua*)	Raw	–	Not mentioned	HS-SPE (sample homogenate)	DB-5 ms	Direct intensity	1	11	(Olafsdottir, Jonsdottir, Lauzon, Luten, & Kristbergsson, 2005)
*Hake*									
Hake (*Merluccius merluccius*)	Raw	–	Wild-caught	HS-SPME (sample homogenate)	DB-5	FD factor	1	11	(Triqui, 2006)
*Mullet*									
Grey mullet (*Mugil cephalus*)	Cooked	Not mentioned	Wild-caught	SDE	DB-Wax	FD factor	1	24	(Cayhan & Selli, 2011)
Red mullet (*Mullus barbatus*)	Raw, steamed, baked	20 min (steaming); 200 °C, 20 min (baking)	Wild-caught	SAFE	DB-Wax	FD factor	3	11	(Salum, Guclu, & Selli, 2017)
*Turbot*									
Turbot (*Scophthalmus maximus*)	Boiled	5 min	Wild-caught	SDE	DB-Wax	FD factor	2	12	(Prost, Serot, & Demaimay, 1998)
Turbot (*Scophthalmus maximus*)	Raw	–	Aquacultured	SDE	BPX-70	Detection frequency	3	23	(Sérot, Regost, Prost, Robin, & Arzel, 2001)

**Abbreviations:** SDE, simultaneous steam distillation-solvent extraction. SAFE, solvent-assisted flavour evaporation. P&T, dynamic headspace purge and trap. HS-SPE, head space-solid phase extraction. HS-SPME, head space-solid phase microextraction.

From this current systematic review of olfactometry data, a total of *n* = 246 volatile compounds were found to be odour-active across 17 saltwater species (raw and cooked) from 46 studies. In a recent review, Jones et al. (2022) reported a total of *n* = 291 odour-active volatile compounds from 31 fresh- and saltwater species across 44 studies. These are a large number of odour-active volatiles in both cases, and the number of compounds identified across studies differed, even within the same seafood species extracted by similar methods (Table 2). For example, a total of *n* = 74 odour-active volatile compounds were reported in rainbow trout (*Oncorhynchus mykiss*) by Mahmoud and Buettner (2017), in contrast to only *n* = 16 by Cengiz, Guclu, Kelebek, and Selli (2023). Such large variations may be explained by differences in the volatile extraction method, the chromatographic separation, and the olfactometric detection method, as well as in the sample characteristics (rearing method and diet, e.g. aquaculture or wild-caught) and cooking conditions (Table 2**).** Although many odour-active compounds can be perceived in GC–O analysis, it should be noted that each volatile has been chromatographically separated, which is quite different to the perception of a complex mixture of volatiles simultaneously. In addition, many of the odours detected by olfactometry have quite low odour activity values (OAVs). Studies have demonstrated that normally only the top odour-impact volatiles (OAV > 1) make a contribution to the sensory characteristics, although there may be exceptions (Grosch, 2001). Flavour recreation and model flavour studies have shown that only a small subset of odour active volatiles are required to recreate a complex odour (Granvogl & Schieberle, 2022). It has been proven that, in general, the odour qualities of most foods can be synthesised or “recreated” through a combination of only about 17% of the most odour-active compounds identified in them (Dunkel et al., 2014). Based on these principles, we shortlisted odour-active volatiles in seafood according to their top 15 ROAs, which were subsequently used in statistical analyses.

**Table 2:** Comparison of mean total fat, saturated fat, protein, carbohydrate, sugar and dietary fibre amongst seafood groups and PBSAs.

	**Total fat** **(%)**	**Saturated Fat** **(g/100** **g)**	**Protein** **(g/100** **g)**	**Carbohydrate** **(g/100** **g)**	**Sugar** **(g/100** **g)**	**Dietary fibre (g/100** **g)**
Crustaceans (*n* = 3)	0.9 b	0.2 b	17.6 ab	0.04 b	0 b	0 b
Molluscs (*n* = 7)	1.2 b	0.3 b	14.6 b	2.2 b	0.1 b	0 b
Oily fish (*n* = 17)	8.4 a	2.1 a	18.7 a	0 b	0 b	0 b
White fish (*n* = 21)	2.8 b	0.7 b	18.1 ab	0.01 b	0.01 b	0 b
Plant-based (*n* = 41)	5.6 a	0.9 b	7.6 c	12.6 a	1.9 a	2.5 a

**Notes**: Seafood macronutrient data were obtained from USDA and FSANZ databases. For each parameter, different letters indicate statistically significant differences (*p < 0.05*) in mean values tested by one-way ANOVA followed by Tukey's post-hoc test.

#### 3.1.1 Odour impact volatiles in raw and cooked seafood

**Fig. 2:**
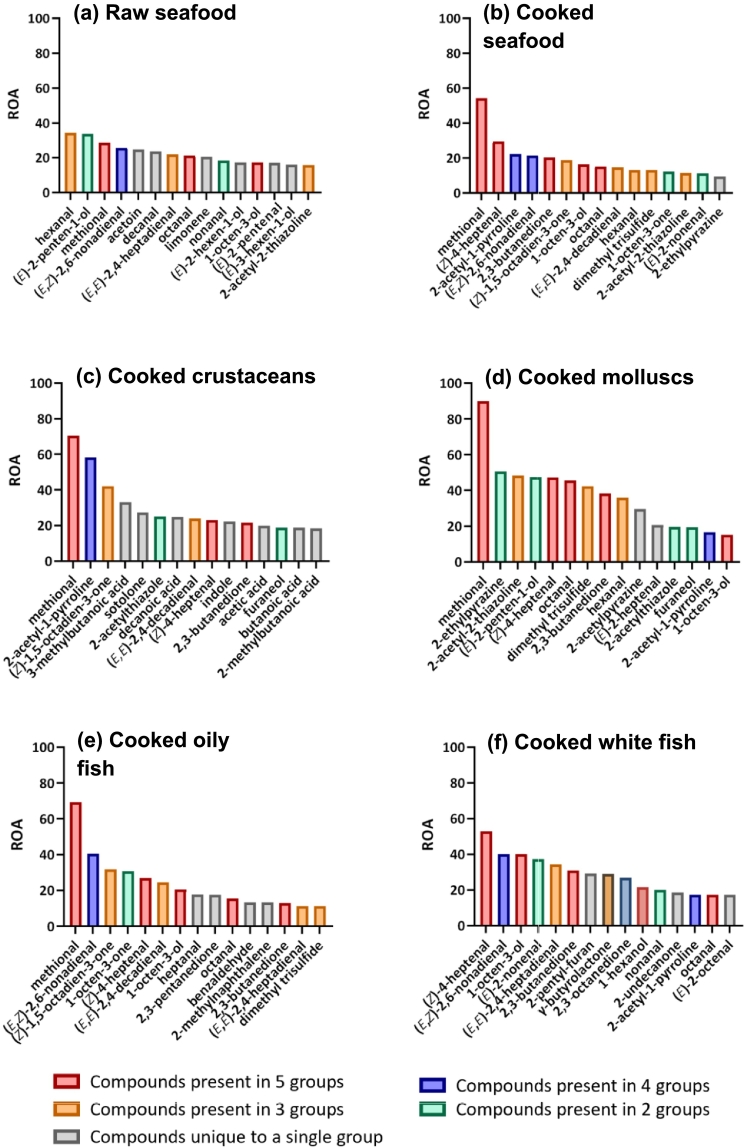
Key volatile profiles of different seafood types. (a) Raw seafood (*n* = 31), (b) cooked seafood (*n* = 28), (c) cooked crustaceans (8), (d) cooked molluscs (*n* = 6), (e) cooked oily fish (*n* = 9) and (f) cooked white fish (*n* = 5). Fifteen odour-active compounds with the highest contribution scores are presented in descending order. Compounds present in 5, 4, 3 and 2 groups are highlighted in red, blue, orange and green, respectively. Compounds unique to a single group are highlighted in grey. (For interpretation of the references to colour in this figure legend, the reader is referred to the web version of this article.)

Fresh seafood has a distinctive and recognisable flavour signature, presumably made up by a group of “core” seafood flavour molecules. Common odour-impact volatile compounds were characterised across both raw (Fig. 2a) and cooked (Fig. 2b) seafood, included; hexanal (*green, grass, fresh*), methional (*cooked potato*), (*E*,*Z*)-2,6-nonadienal (*cucumber, floral, green*), octanal (*citrus, sweet, orange*), 1-octen-3-ol (*mushroom, fermented, potato*) and 2-acetyl-2-thiazoline (*roasted, popcorn-like, nutty*), however, their relative influence differed, as reflected by their ROAs.

Impact volatiles that were exclusively associated with raw seafood flavour, included; (*E*)-2-penten-1-ol (*mushroom, fish-like, marine*), 3-hydroxy-2-butanone (acetoin) (*buttery, green*), decanal (*green, fresh, floral*), (*E*,*E*)-2,4-heptadienal (*fatty, green, mushroom*), limonene (*pine, floral, fresh*) and (*E*)-3-hexen-1-ol (*moss, green, fresh*). Most of these are derived from the oxidation of unsaturated lipids, aldehydes and alcohols (Liu et al., 2020). Acetoin is a result of microbial activity and limonene accumulates through dietary intake from marine biota (Mahmoud & Buettner, 2017). Interestingly, 2-acetyl-2-thiazoline, which is usually formed during thermal processing, was also amongst the top 15 odour impact volatiles in raw seafood. Its presence is likely attributed to both lipid oxidation and microbial activity prior to cooking but may also be a thermal artefact resulting from the extraction method. Surprisingly, TMA, the highly volatile compound most often associated with typical fishy odour in the literature, was not in the top 15 odour-impact volatiles in raw seafood. We speculate that only very fresh seafood was selected for these studies and TMA may have been present at very low concentration or lost during flavour extraction for olfactometry studies, hence its olfactory contribution may be underestimated.

Unsurprisingly, a different combination of odour-impact volatiles was important for generic cooked seafood, mainly derived from the thermal degradation of unsaturated fatty acids and amino acids. These included (*Z*)-4-heptenal (*fish-like, potato, biscuit*), 2-acetyl-1-pyrroline (*roasted, popcorn-like*), 2,3-butanedione (*butter, caramel, sweet*), (*Z*)-1,5-octadien-3-one (*metallic, geranium-like*), hexanal (*green, grass, fresh, garlic*) and 2-ethylpyrazine (*nutty*) (Nieva-Echevarría et al., 2017).

#### 3.1.2 Discrimination of seafood varieties based on odour-impact compounds in raw seafood

**Fig. 3:**
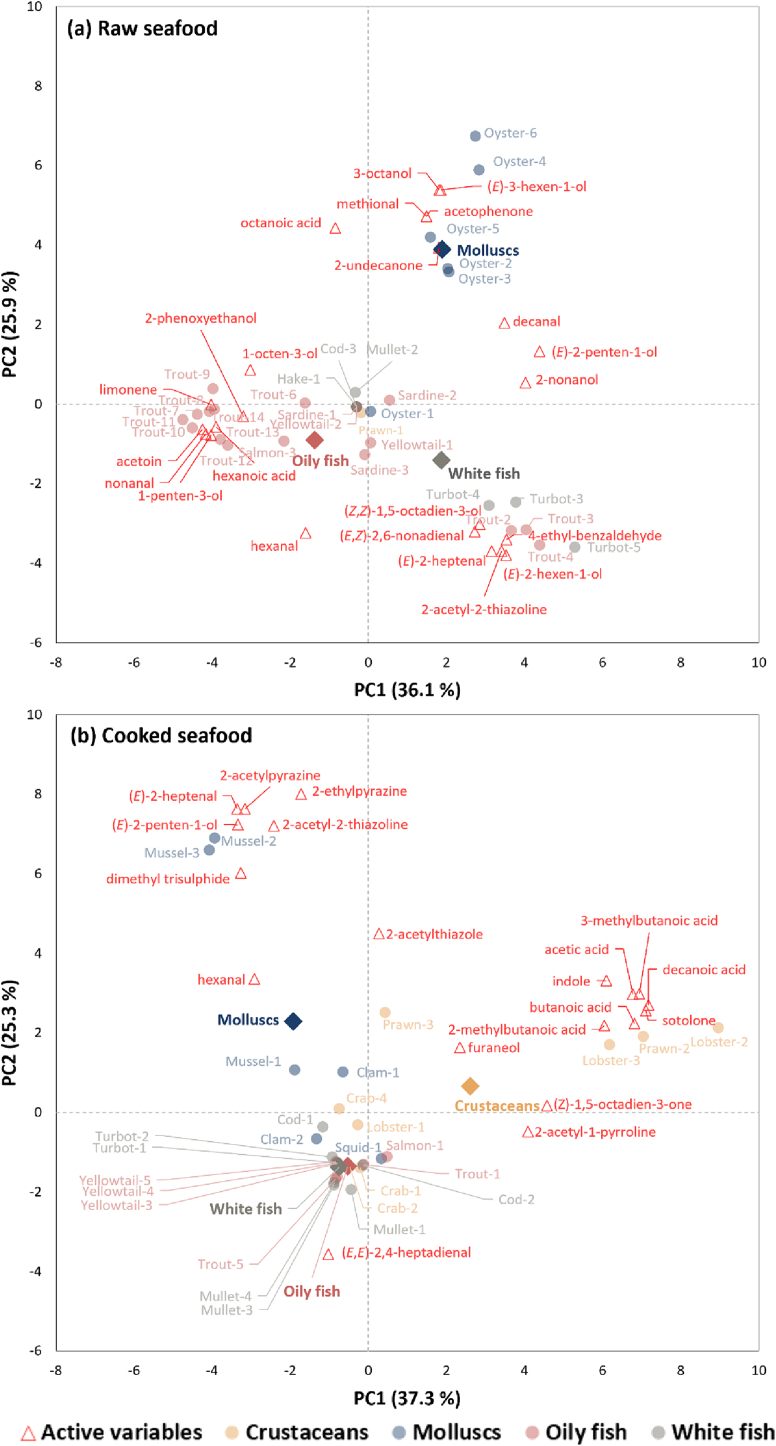
PCA biplots illustrating distribution of (a) raw and (b) cooked seafood species based on key odour active compounds. Loadings of volatile compounds are highlighted in red triangles. Samples from crustaceans (*n*_raw_ = 1, *n*_cooked_ = 8), molluscs (*n*_raw_ = 6, *n*_cooked_ = 6), oily fish (*n*_raw_ = 18, *n*_cooked_ = 7) and white fish (*n*_raw_ = 6, *n*_cooked_ = 7) groups are labelled in gold, blue, pink and grey, respectively. Group centres are labelled with “⯁”. (For interpretation of the references to colour in this figure legend, the reader is referred to the web version of this article.)

To further understand differences in the odour signatures of different seafood species, PCA biplots were generated. For raw seafood (*n* = 31, Fig. 3a), 62.0% of the total variance was explained by the first two PCs. Oily fish were mainly located on the left-hand side of PC1 and were associated with the lipid derived volatiles, nonanal (*marine, geranium, plastic*), 1-penten-3-ol and 1-octen-3-ol as well as hexanoic acid, acetoin, limonene and 2-phenoxyethanol. The oyster samples were differentiated on PC2, mainly by (*E*)-3-hexen-1-ol, 3-octanol (*moss, sulphur*), methional, acetophenone (*animal, grass, nutty*) and 2-undecanone (*fruity, fresh, cucumber*). Pennarun, Prost, and Demaimay (2002) previously reported that (*E*)-3-hexen-1-ol was important to the overall odour of raw oyster imparting a pleasant fresh odour quality. Three turbot samples (white fish) and three trout samples (oily fish) were characterised by the strong odour contribution of (*E*)-2-penten-1-ol, (*Z*,*Z*)-1,5-octadien-3-ol (*cooked, moss, mushroom*), 2-acetyl-2-thiazoline, (*E*)-2-heptenal (*grass, roasted, fatty*), (*E*,*Z*)-2,6-nonadienal, methional and 4-ethyl-benzaldehyde (*anise, fruity, marine*), forming a cluster in the negative side of PC2. Three white fish samples (Hake-1, Cod-3, Mullet-2), and Oyster-1 and Sardine-2 (oily fish) were found around the origin.

Although most trout samples (oily fish) were clustered on the left of PC1, some were closer to the turbot (whitefish) samples. This was not surprising, as the trout samples in this study were subject to varying diets and aquatic environments (Table 1). Trout samples 2–4 were fed ad libitum on diets containing different types of lipids and reared in cages in an experimental farm (Sérot, Regost, & Arzel, 2002), whereas trout samples 6–14 were fed with a standard commercial feed and reared with the same commercial production procedures in different commercial farms (Cengiz et al., 2023). This variation underscores the significant impact of aquacultural strategies on the aroma profile of the end product.

#### 3.1.3 Discrimination of seafood varieties based on odour-impact compounds in cooked seafood

The PCA biplot (*n* = 28, Fig. 3b) based on the top odour-impact volatiles in cooked seafood, explained (62.6%) of total variance, with similar discrimination on PC1 and PC2. Mollusc samples (mussel, clam, squid) were located on the top-left and associated with a relatively high concentration of 2-acetylpyrazine (*burnt, grilled, nutty*), (*E*)-2-heptenal, (*E*)-2-penten-1-ol, 2-acetyl-2-thiazoline, 2-ethylpyrazine, and dimethyl trisulphide (*cabbage, sulphur, garlic*). Two mussel samples (Mussel-2, Mussel-3) were especially high in these compounds. The crustacean samples were predominantly located on the right side of PC1, strongly driven by a range of volatile organic acids, including 2-acetyl-1-pyrroline, 3-methylbutanoic acid, acetic acid, decanoic acid, butanoic acid as well as indole (*mothball-like*) and sotolone (*seasoning-like*). Most of oily fish and white fish were associated with high concentration of (*E*,*E*)-2,4-heptadienal and located on the negative side of PC2. However, surprisingly, a clear separation between these two groups was not observed.

In general, the PCA discrimination of cooked seafood was less clear than for raw. A greater similarity in the key volatile profiles amongst different types of cooked seafood, was indicated by the clustering of oily fish, white fish and crustaceans near the origin. This was largely due to the prevalence of common thermally-generated compounds across the cooked seafood species. Despite the similarities amongst cooked seafood samples in the PCA, it is widely acknowledged that each type of seafood has its own distinct aroma and flavour characteristics when consumed. Since most seafood undergoes heat treatment before being eaten (oysters and fish sashimi being exceptions), the key odour-active compounds of cooked seafood for each group were examined by the same approach.

### 3.2 Key volatile profiles of different types of cooked seafood

Key volatile profiles of the four types of cooked seafood exhibit certain similarities (Fig. 2c-f), which is in line with the findings in PCA (Fig. 3b). Both (*Z*)-4-heptenal and 2,3-butanedione were important odour-impact volatiles for all cooked seafood types, with (*Z*)-4-heptenal possessing the highest ROA in white fish. Although methional was not in the top 15 list of white fish (Fig. 2f), it was the most potent odour-active compound for crustaceans (Fig. 2c), molluscs (Fig. 2d) and oily fish (Fig. 2e). Similarly, 2-acetyl-1-pyrroline was crucial to all cooked seafood types except oily fish, ranking with the second-highest score in crustaceans. Together with other thermally generated compounds including (*Z*)-1,5-octadien-3-one, dimethyl trisulphide, 2-acetyl-2-thiazoline, furaneol (*caramel, strawberry, sweet*), 1-octen-3-ol and some aldehydes that are commonly found across different types of seafood, it is reasonable to suggest that these are key volatile components for forming the fundamental aroma profile of cooked seafood. Beyond these generic seafood volatiles, the addition of distinct odour-active compounds within each group are responsible for the unique flavour characteristics of different types of seafood. These compounds are discussed in the following subsections.

#### 3.2.1 Crustaceans

As shown in the result (Fig. 2c), sotolone, indole and a range of organic acids including 3-methylbutanoic acid (*butter, cheese, fermented*), decanoic acid (*musty*), acetic acid (*vinegar*), butanoic acid (*sweaty*) and 2-methylbutanoic acid (*cheese, pungent*) were uniquely influential to the aroma profile of cooked crustaceans. Jones et al. (2022) suggested that these acid compounds make a limited contribution to the odour characteristic of seafood. Furthermore, based on the reported frequencies of “crustacean-like” odour in GC–O studies, the authors summarised five key volatiles, which included (*Z*)-4-heptanal, trimethylamine, dimethyl sulphide, isopropyl methyl disulphide, methyl-2-methyl propyl disulphide and 1-pyrroline. From our review, we conclude that only (*Z*)-4-heptenal plays a role in crustacean aroma; the remaining four compounds were not found to be key odour-impact volatiles of cooked crustaceans in this present result.

This suggested that the subtle aroma characteristic of crustaceans may be shaped by a combination of odour-active compounds with diverse odour qualities rather than relying on a single molecule with crustacean-like odour. It is also noteworthy that none of the GC–O studies identified bromophenols as odour impact volatiles in crustacean flavour, despite previous research showing their importance in fresh wild crustacean flavour (Whitfield, Helidoniotis, Shaw, & Svoronos, 1998; Whitfield, Helidoniotis, & Smith, 2002). Many studies have highlighted the importance of brominated compounds to authentic seafood flavour, including finfish. Future research is needed to confirm contributions of these volatile compounds to cooked crustaceans and other species.

#### 3.2.2 Molluscs

Maillard generated compounds with roast nutty attributes including 2-ethylpyrazine, 2-acetyl-2-thiazoline, and 2-acetylpyrazine were exclusively found in the volatile profile signature of cooked molluscs (Yu et al., 2021). As shown in Table 2, molluscs (*n* = 9), contained the highest total sugar content amongst seafood types potentially enhancing the thermal production of pyrazines. (*E*)-2-Penten-1-ol is the fourth highest odour-impact compound and has been associated with mushroom, fish-like, marine and green odour qualities. While there is no direct evidence of a metabolic synthetic pathway in molluscs, it is documented that seaweed is a rich source of (*E*)-2-penten-1-ol (López-Pérez, Picon, & Nuñez, 2017). Considering seaweed forms a major part of the diet for molluscs, especially shellfish (Baumgartner, Motti, de Nys, & Paul, 2009), it is likely that this compound accumulates in molluscs through dietary intake. Additionally, two odourants with green odour notes, hexanal and (*E*)-2-heptenal were unique to the key volatile profile of cooked molluscs.

#### 3.2.3 Finfish

Oily fish had a significantly higher fat content than white fish (Table 2), leading to the expectation of more pronounced lipid oxidation during cooking, and consequently, a greater variety of oxidation products. Interestingly, the results (Fig. 2e**&f**) indicated that the key volatile profiles of both types of cooked fish were largely made up of aldehydes and ketones associated with lipid oxidation (Jones et al., 2022). Despite this similarity, distinct aroma characteristics of two types of cooked fish were identified, attributed to the specific odour qualities of individual compounds. In oily fish (Fig. 2e), three earthy compounds including 1-octen-3-one (*mushroom, earthy, egg*), heptanal (*green, floral, earthy*) and 2-methylnaphthalene (*earthy, plastic, green*) played a significant role in the aroma profile, together with 2,3-pentanedione (*butter, caramel, malt*) and benzaldehyde (*nutty, roasted, fruity*) which contributed to the sweet odour characteristics. On the other hand, the volatile profile of white fish (Fig. 2f) was primarily shaped by fish-like odourants including (*E*)-2-nonenal (*earthy, green, fish-like*), 1-hexanol (*green, alcohol, fish-like*), nonanal and octanal. This aroma profile is complemented by compounds with diverse odour qualities, including 2-pentylfuran (*beany, earthy, metallic*), γ-butyrolactone (*oily*), 2,3-octanedione (*green*) and 2-undecanone, which together likely create a distinct volatile spectrum compared to oily fish.

### 3.3 Other odour-active compounds in seafood

Importantly, some odour-active compounds that do not normally play a role in seafood flavour profiles, may occasionally be present at concentrations above sensory thresholds. For example, geosmin (GSM) and 2-methylisoborneol (MIB)—metabolites produced by aquatic bacteria such as cyanobacteria and actinobacteria, as well as fungi, —are known for imparting musty and earthy odours into seafood and other aquatic creatures (Lindholm-Lehto, Koskela, Kaseva, & Vielma, 2020; Smith, Boyer, & Zimba, 2008). As these compounds are hydrophobic, they accumulate in fatty tissues of fish (Lindholm-Lehto et al., 2020), and are commonly found in aquacultured seafood (Smith et al., 2008). In agreement, across our investigated GC–O studies, MIB and GSM were only detected in aquacultured prawn (Mall & Schieberle, 2016), barramundi (Frank, Poole, Kirchhoff, & Forde, 2009), salmon (Guo et al., 2021) and trout (Mahmoud & Buettner, 2017). As aquacultured seafood becomes more available to consumers (farmed barramundi & salmon), opportunities for exposure to muddy flavour increases. While extreme muddy flavour is considered a taint, in some circumstances, and at low concentration it may actually be desirable.

Furthermore, while TMA can impart a pleasant crustacean-like odour at low concentrations, it becomes unpleasantly fishy at higher concentrations (Cadwallader, Tan, Chen, & Meyers, 1995). However, almost half of the seafood samples from the reviewed GC–O studies were heat-treated prior to analysis. Considering the low boiling point of TMA (2.87 °C at 760 mmHg) (National Center for Biotechnology Information, 2024), which makes it extremely volatile when exposed to heat, it is reasonable to speculate that TMA is lost during cooking. In studies using raw samples, the samples were kept fresh until analysis. As a result, TMA exhibited an extremely low ROA in this study.

### 3.4 A lack of GC–O studies on plant-based products

Our systematic review of GC–O studies demonstrated distinctive volatile profiles for various seafood types. These insights could serve as benchmarks for comparing the odour qualities between authentic seafood species and be used to design better flavours for PBSAs. Recent research has explored potential volatile compounds in plant-based ingredients, such as microalgae species and seaweeds with the aim of providing guidelines for PBSA flavour formulation (Coleman et al., 2022; Moreira, Ferreira-Santos, Teixeira, & Rocha, 2022). There are few published GC–O studies on PBSAs or on plant-based ingredients for PBSAs; it would be useful for confirming the actual olfactory qualities.

### 3.5 Non-volatile profiles of seafood and plant-based alternatives

**Fig. 4:**
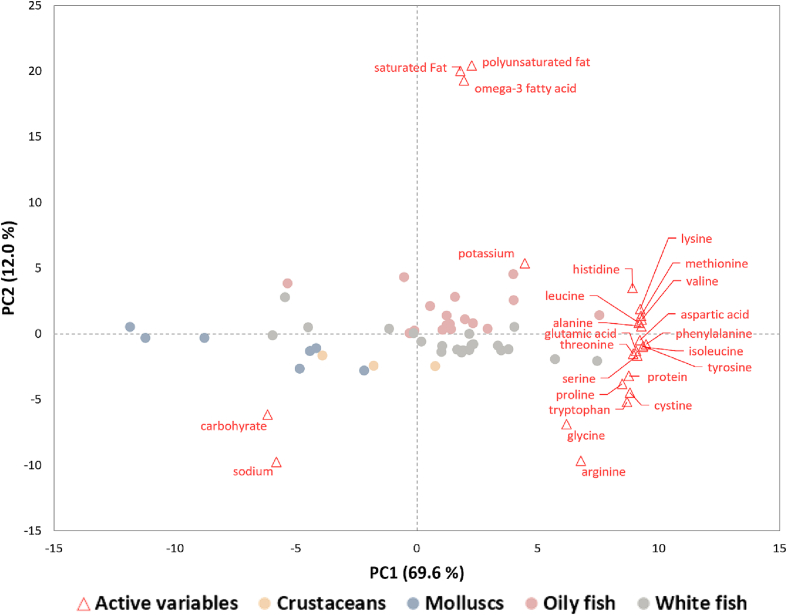
PCA biplots illustrating distribution of seafood species based on their contents of non-volatile compounds. Loadings of non-volatile compounds are highlighted in red. Samples from crustaceans (*n* = 3), molluscs (*n* = 7), oily fish (*n* = 17) and white fish (*n* = 21) groups are labelled in gold, blue, pink and grey, respectively. (For interpretation of the references to colour in this figure legend, the reader is referred to the web version of this article.)

Non-volatile tastants, including free amino acids, nucleotides, and minerals, directly impart taste qualities (Sarower et al., 2012). Macronutrients including fats, proteins, sugars and fibre not only act as flavour precursors, being transformed by heating to produce an array of odour-active volatile compounds, but also influence the release of flavour volatiles through intermolecular interactions (Tournier, Sulmont-Rossé, & Guichard, 2007). Hence, macronutrient composition can offer logical insights into the complex spectrum of seafood flavour. The PCA biplot (Fig. 4) of non-volatile compounds of different seafood groups illustrates typical variation.

#### 3.5.1 Amino acids, nucleotides and organic acids

The amino acid data represent total amino acids, encompassing both free and bound forms, however amino acids directly contribute to taste only in their free form. Due to a lack of reliable published free amino data, we have relied solely on database total amino acid values, although we acknowledge this limitation.

As shown in Fig. 4, amino acids were key factors differentiating our investigated seafood in PC1. Molluscs and two out of three crustacean species were distinguished from other groups primarily due to their relatively low amino acid (and total protein) content. A wider variation in amino acids were measured across white and oily fish species. Free glutamic and aspartic acids are well-known for their contribution to umami taste (Shallenberger, 1993). Arginine, known for its sweet taste, was associated with some white fish samples on the negative side of PC2. While other amino acids may also impart subtle taste qualities, they are often present in seafood at concentrations below their taste thresholds. For example, only glutamic acid, glycine and arginine were found to be taste-active in cooked crab (Chen & Zhang, 2007) and raw oyster (Bi et al., 2021). In the absence of reliable data on free amino acid profiles for these species any general conclusions on flavour profiles is speculative.

IMP and GMP are important taste-active nucleotides that contribute to the umami taste of seafood (Sarower et al., 2012). Organic acids such as succinic acid also contribute to the taste of seafood (Bi et al., 2021; Sarower et al., 2012). However, due to the lack of availability of accurate nucleotide and organic acid data in nutrient databases, no corresponding data are included in this review.

#### 3.5.2 Minerals

Compared to terrestrial meats, seafood is a richer source of minerals such as sodium, potassium, calcium, magnesium, iron, copper and zinc (Tacon, 2023). Minerals typically impact salty and/or bitter tastes. Sodium and potassium are the only minerals reported at levels above their corresponding taste threshold (Chen & Zhang, 2007; Rotzoll et al., 2006). Therefore, sodium and potassium were included in the PCA in this present study. Sodium concentration was highest in molluscs and crustaceans, followed by white and oily fish. The reverse order was found for potassium. In addition to contributing to saltiness, sodium acts synergistically with glutamic acid (and others to a lesser extent) to enhance umami taste (Lioe et al., 2005). Therefore, the higher levels of sodium found in crustaceans and molluscs may lead to a richer more complex taste profile, rather than simply equating to a more pronounced salty taste.

#### 3.5.3 Matrix composition

The macronutrient composition of the food matrix acts as a reservoir of flavour compounds and also affects the oral breakdown and flavour release, crucial in understanding the sensory potential of food. Differences in the amount of fat, protein and carbohydrate, affect the flavour potential and release (Frank, Appelqvist, Piyasiri, & Delahunty, 2012; Frank, Appelqvist, Piyasiri, Wooster, & Delahunty, 2011). Differences in macronutrient composition of seafood and commercially available PBSAs (*n* = 41) are summarised (Table 2 and the PCA biplot (Fig. 4)).

The samples were mainly separated on PC1 (69.6%) based on different total protein and AA concentration; white and oily fish had higher protein and AA content than crustaceans and molluscs. The sodium content was higher in molluscs and crustaceans. Oily fish were discriminated from other seafood on PC2, mainly driven by higher saturated fat, polyunsaturated fat and omega-3 fatty acid content. This is also in line with the comparison in Table 2. Fats, particularly unsaturated fatty acids, play a significant role in aroma development, primarily through lipid oxidation (Nogueira et al., 2019). Therefore, volatile profiles of oily fish species such as salmon (Varlet, Knockaert, Prost, & Serot, 2006), sardine (Ganeko et al., 2008) and trout (Sérot et al., 2002) are typically more abundant in lipid oxidation products like compared to other seafood groups. For PBSAs, the average total fat content was not significantly different from that of oily fish (Table 2). However, considering that PBSAs have a considerably lower saturated fat content, the primary distinction in fat composition between oily fish and PBSAs is attributed to unsaturated fats. In addition to the difference in fat levels, the types of fats used in PBSAs, which are derived from plant oils, are different from those in authentic seafood, known for being rich in long-chain omega-3 fatty acids. This difference in fat composition can lead to a distinct profile of aroma compounds. For example, compounds like (*E*,*E*)-2,4-heptadienal and (*E,E*)-3,5-octadien-2-one, typically resulting from EPA and DHA oxidation and frequently found in seafood (Nogueira et al., 2019), were not detected as aroma-active in plant-based alternative products (Zhang et al., 2023).

Furthermore, given the inherent differences between the high protein and low carbohydrate nature of seafood and the low protein and high carbohydrate nature of PBSAs (Table 2), the efficacy of Maillard reaction and the production of volatiles associated with the reaction is likely to vary.

### 3.6 Aroma and flavour sources used in plant-based seafood alternatives

**Fig. 5:**
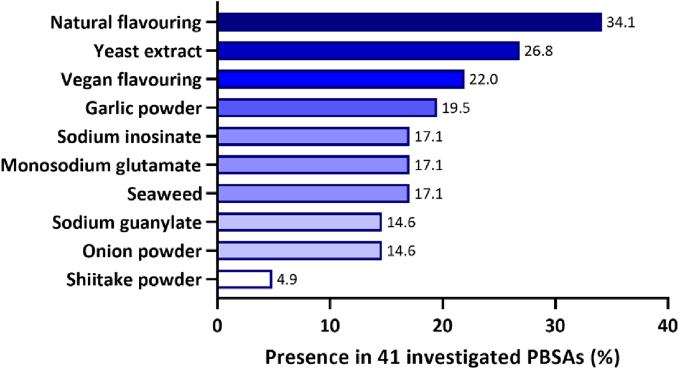
Flavouring agents in 41 investigated commercial PBSAs. A darker-coloured bar indicates a higher frequency of presence.

An appealing flavour profile is an important characteristic of any successful food product. In the formulation of PBSAs, emulating subtle seafood aromas and flavours requires the careful addition of flavour molecules in an appropriate food matrix. Based on our investigation of the ingredient lists of 41 commercially available PBSAs, sodium inosinate, glutamic acid (or monosodium glutamate (MSG)), and sodium guanylate are the most used single-substance flavour enhancers, shown in Fig. 5 and dataset in **Supplementary Data File 1**. Although these substances effectively impart a savoury taste to the product, achieving a more complex flavour profile that closely resembles seafood may require the inclusion of additional flavouring agents. While the exact compositions of ‘natural flavouring’ and ‘vegan flavouring’ as claimed on labels remain unclear, yeast extract, garlic powder, onion powder, seaweed and shiitake powder are also common plant-based flavourings high in umami compounds used in the investigated PBSAs.

#### 3.6.1 Yeast extracts

Due to their rich content of flavour precursors, such as free amino acids, nucleotides, vitamins, fats and sugars, as well as odour-active volatile compounds, yeast extracts have been widely used for enhancing meaty characteristics of food products for decades (Ames & Leod, 1985). Despite the variation in volatile profiles of different yeast extracts (Jones et al., 2022; Lin et al., 2014), many common odour-active volatiles in yeast extracts are also found in seafood. Compounds such as 3-methylbutanal, hexanal, and nonanal, which are abundant in yeast extracts, can contribute to fish-like aroma (Jones et al., 2022; Lin et al., 2014). Furthermore, dimethyl sulphide, characterised by its crustacean-like note, has been identified in some yeast extract products (Wang et al., 2019).

#### 3.6.2 Garlic and onion powder

Similar to yeast extracts, garlic and onion powder contains a ranges of aldehydes (3-methylbutanal, hexanal, octanal, nonanal and (*E*)-2-octenal), and savoury sulphur compounds (dimethyl sulphide and dimethyl disulphide) and furans (2-pentylfuran) that can mimic seafood aromas (Li, Zhan, Tian, Wang, & Ji, 2021; Villière et al., 2015). In addition to direct provision of odourants, onion is proven capable of masking undesired fishy flavour notes (Bedrníček et al., 2020). While not reported in seafood studies, it is a well-recognised practice amongst food manufacturers to use garlic powder to mask unwanted flavours, which is evidenced by the frequent inclusion of garlic powder in the ingredient lists of the investigated PBSAs (Fig. 5). This is a property particularly advantageous in PBSAs formulated with marine ingredients, where fishy aromas may be overly pronounced. Additionally, for all plant-based alternatives, where legumes are commonly employed as protein sources, mitigating the inherent beany flavour is essential to ensure these analogues closely resemble their seafood counterparts. In this context, garlic and onion powder can play a useful role in flavour modification.

#### 3.6.3 Seaweed and algal oils

Some PBSAs incorporate microalgae oil, however, its inclusion is primarily for nutritional supplementation of omega-3 fatty acids, which are also an important source of seafood odour impact volatiles. Therefore, seaweed (e.g. tangleweed (*Laminaria digitata*)), also known as macroalgae, is the only marine ingredient for flavouring purposes found amongst our investigated PBSAs. From a volatile composition perspective, seaweed encompasses many of the important identified odour-active seafood odourants, such as (*Z*)-4-heptenal, (*E*,*E*)-2,4-heptadienal, 1-octen-3-ol and dimethyl sulphide (Jones et al., 2022). The ocean environment is also a rich source of halogens - chlorine, bromine and iodine. As a result of enzymatic halogenation, algae can contain a high concentration of organobromine and organoiodine compounds such as dibromoethane, iodoethane, iodopentane, dibromochloromethane, bromodichloromethane and chloroiodomethane (Dong et al., 2020; Laturnus, Wiencke, & Klöser, 1996). Additionally, bromophenols have been widely reported in marine biota and seafood species (e.g., 2-bromophenol, 4-bromophenol, 2,4-dibromophenol, and 2,4,6-tribromophenol) (Chung, Ma, & Kim, 2003; Dong et al., 2020). Many studies have shown that bromophenols are important in fresh wild seafood flavour, but often absent in aquaculture seafood produce (Chung et al., 2003; Whitfield et al., 2002). Given their appealing marine odours and extremely low odour thresholds (Fuller, Frank, Fitzhenry, Smyth, & Poole, 2008; Whitfield, Last, Shaw, & Tindale, 1988), bromophenols could significantly enhance flavour properties of PBSAs. To the best of our knowledge, bromophenols have not been identified in other common food plants. In this regard, certain seaweed species, or their extracts, may be ideal for the natural fortification of bromophenols in PBSA formulation.

Surprisingly, despite the extensive published literature showing the importance of bromophenols to natural seafood flavour, no non-targeted GC–O studies have conclusively demonstrated their contribution to volatile profiles with validated reproducibility and repeatability. This may be due to the instrumental detection limit constraints of non-targeted analysis. Therefore, no quantitative GC–O results of bromophenols have been reported in our systematic literature search and their role in discriminating seafood samples remain unclear.

#### 3.6.4 Mushroom powder

Fungi, including dried mushrooms, are rich sources of umami taste amino acids and 5′-nucleotides, and have been commonly used as taste enhancer for producing various food products (Harada-Padermo, Dias-Faceto, Selani, Conti-Silva, & Vieira, 2021). Amongst our investigated PBSAs, although only 4.9% of them label the use of shiitake powder, however the actual number of products containing mushroom ingredients is likely higher, as mushroom extracts are often declared as “natural” and vegan flavourings.

Volatile profiles of different mushrooms are highly diverse. For example, limonene is the key odour-active compounds in straw mushroom (*Volvariella volvacea*) (Mau, Chyau, Li, & Tseng, 1997), and sulphur compounds, such as dimethyl sulphide and dimethyl disulphide are potent odourants of shiitake (*Lentinus edodes*) (Zhou et al., 2020) and black truffle (*Tuber indicum*) (Splivallo & Ebeler, 2015). On the other hand, 3-octanone, 1-octen-3-one, 3-octenol and 3-octanol are commonly found amongst mushroom species (Zhu et al., 2022). The compounds discussed above were also found important for building the flavour profiles of a range of seafood.

## 4 Challenges in balancing flavours in PBSA formulation

Anecdotally, most PBSAs have quite strong flavours compared to fresh seafood. In general, the flavour profiles of PBSAs do not change much from raw to cooked. Variation in odour qualities of volatiles at different concentrations is a crucial aspect to consider. Laing, Legha, Jinks, and Hutchinson (2003) demonstrated that heptanal, which is also widely found in seafood, is described as “fragrant”, “cucumber”, “cool”, “light” and “malty”, but at higher concentrations, its odour quality shifts to “oily”, “sickening”, “dirty linen”, “heavy”, and “bean-like” notes. (Laing et al., 2003). This effect has also been observed in studies based on different food matrices. In pea-protein-based products, compounds such as hexanal, 2-nonanone, 1-penten-3-ol and 2-pentylfuran have been identified as the primary off-flavour compounds due to their overwhelming beany and green notes (El Youssef et al., 2020). However, at lower concentrations typically found in seafood, these compounds were the key odourants building desirable seafood flavour profiles of various species, such as prawn (Mall & Schieberle, 2016; Okabe, Inoue, Kanda, & Katsumata, 2019), crabs (Yu & Chen, 2010), salmon (Guo et al., 2021; Milo & Grosch, 1996), trout (Milo & Grosch, 1995; Selli, Rannou, Prost, Robin, & Serot, 2006) and oysters (Pennarun et al., 2002; Pennarun, Prost, Haure, & Demaimay, 2003; Piveteau et al., 2000). Although research on solid food matrices is limited, evidence from model solutions indicates that interactions of odourants can significantly affect the overall perception of odour quality. The synergistic effect of aroma compounds, which refers to the enhancement of odour intensity to a degree that exceeds the combined effect of the individual compounds, has been proven by many studies (Cameleyre, Lytra, Tempere, & Barbe, 2015; Culleré, Cacho, & Ferreira, 2007; Fuente-Blanco, Saenz-Navajas, & Ferreira, 2016).

In addition to the uncertainties associated with combining odours in mixtures, the effect of food matrix components on flavour release is an important aspect to consider. It has been well-established that overall fat content is a strong determinant of aroma release due to its impact on lipophilic volatiles in the food matrix (Frank et al., 2011; Frank et al., 2012). Numerous studies have demonstrated that even minimal changes in fat content can significantly impact the release of volatiles, particularly affecting non-polar compounds. For example, Schirle-Keller, Reineccius, and Hatchwell (1994) reported that a 1% increase in oil content in the oil/water model solution resulted in an over 85% decrease in the detected non-polar volatiles limonene and ethyl heptanoate in the headspace. In addition, volatility of odourants is highly susceptible to the length of the fatty acid chains and the level of unsaturation in the triglycerides. Harvey, Druaux, and Voilley (1995) demonstrated that the oil/water partition coefficients of ethyl butanoate and 2,5-dimethylpyrazine were significantly lower in emulsions containing tributyrin (triacylgycerol of butanoic acid) compared to those with triolein (triacylglycerol of oleic acid). As shown in Table 2, there exists a significant difference in fat contents amongst seafood groups and PBSAs. Additionally, given the variation in types of fat found in seafood and PBSAs, the partitioning of volatile flavour compounds may vary significantly.

Seafoods lack dietary fibre in contrast to PBSAs (Table 2). Although fibre is not directly involved in aroma formation, it can inhibit aroma release by forming a diffusion barrier that entraps volatiles within the matrix. The retention effect of oligofructose on aroma compounds including benzaldehyde, ethyl butanoate and butyl isovalerate has been proven in vitro with proton-transfer-reaction mass spectrometry (PTR–MS) by Siefarth et al. (2011). Later, such effect has been confirmed by in vivo PTR-MS and sensory evaluations (Muñoz-González, Brule, Martin, Feron, & Canon, 2021). Apart from fibre, there are also significant differences in other major components between authentic seafood and PBSAs. It is clear that proteins (Tromelin, Andriot, & Guichard, 2006), polysaccharides (Jouquand, Ducruet, & Giampaoli, 2004) and fats (Ayed, Martins, Williamson, & Guichard, 2018) can supress the release of aroma compound due to hydrophobic interactions, hydrogen bonding, or even ionic interactions. Hence, it is reasonable to speculate that the higher total fat, carbohydrate, and sugar contents, along with lower protein levels in PBSAs (Table 2), may result in a significantly different volatile retention effect compared to authentic seafood.

Furthermore, taking into account the masking effect of aromas, as discussed in Section 3.5.2, the formulation of flavours, even when based on individual substances, is far from straightforward. Consequently, the challenge lies in determining the appropriate quantities and optimal combinations of flavouring ingredients that carry these odourants, to successfully recreate the desired seafood flavour. Preferably, flavour formulation is guided by trained flavourists and sensory panels.

## 5 Conclusions and future perspectives

In the increasingly competitive alternative protein market, improving the aroma and flavour quality is key to increasing consumer acceptance, especially for PBSAs. Through a systematic review of published GC–O data on seafood and subsequent statistical analysis, we confirmed many odour-active compounds that may be essential for authentic seafood flavour and also differentiate between raw and cooked seafood, species. Generally, raw seafood exhibits more distinct key volatile profiles compared to those of its cooked state. In crustacean samples, a variety of volatile acid compounds such as 3-methylbutanoic acid, acetic acid and decanoic acid, along with sotolone and indole, were crucial to the core volatile profile. Cooked molluscs were typically distinguished by nutty compounds such as 2-ethylpyrazine, 2-acetyl-2-thiazoline, and 2-acetylpyrazine, which are products of the Maillard reaction. Although oily fish and white fish shared similarities in their key volatile profiles which are dominated by lipid oxidation products, unique odourants specific to each group were found. Earthy and sweet odourants are prominent in cooked oily fish, whereas cooked white fish is shaped by more typical “fish-like” odourants, including (*E*)-2-nonenal, 1-hexanol, nonanal, and octanal. A comparison of the macronutrient compositions between authentic seafood and commercially available PBSAs underscored significant differences. Currently, different natural flavouring agents have been used in the production of PBSAs. Their plant-based nature and diverse volatile compositions including those with unique characteristics, such as seaweed, offer strong theoretical fundamentals for more successful formulation seafood-like plant-based products.

Furthermore, alga and extracts stand as the sole marine-based ingredients used in PBSA formulations, such as in vegan shrimps, fish broth, and caviar (Coleman et al., 2022). As discussed, theirinherent marine taste and rich content ofmarine volatiles make them ideal candidates. However, an exclusive reliance on algae may limit the flavour authenticity, underscoring the need for further research into other potential flavouring agents. Therefore, exploring novel non-animal flavouring ingredients is of importance, such as those derived from fungi or fermentation. Nut seeds, for instance, possess an array of volatile compounds deemed critical for differentiating various seafood varieties. Notably, hazelnut contains 2,3-pentanedione, hexanal, heptanal, 2-pentylfuran, 1-octen-3-one and acetic acid (Alasalvar, Shahidi, & Cadwallader, 2003; Fischer et al., 2017), which were found essential for raw and various types of cooked seafood in our results. Moreover, nut seeds can be a good sources of polyunsaturated fatty acids including different types of omega-3 (Maguire, O'sullivan, Galvin, O'connor, & O'brien, N., 2004) and proteins (Mandalari et al., 2021), important substrates in lipid oxidation and Maillard reactions.

Moreover, flavour improvement can also be achieved by adopting other processing methods, such as roasting (Alasalvar et al., 2003), fermentation (Rodriguez-Campos et al., 2012) and enzyme treatments (Zhao et al., 2020). These methods can effectively increase the levels of desirable flavour substances as well as generate new compounds, further expanding the flavour reservoir for PBSA formulation.

It is important to recognise some limitations in this review. Firstly, the number of available GC–O studies is limited, meaning the sample sizes in each group may not adequately represent the entire population (Table 2**)**. Secondly, the reviewed GC–O studies have different methods and sample conditions. These factors may introduce bias into the statistical analysis. Thirdly, in GC–O analysis, compounds are separated prior to perception, which does not account for the aroma and flavour interactions that occur during actual food consumption. Lastly, the cooking methods employed in the GC–O studies in this review (steamed, boiled) may differ significantly from those typically used in everyday cooking (grilled, fried etc.), resulting in variations in the aroma and flavour profiles. Given these limitations, a systematic study on authentic seafood and PBSAs, assessing both volatile and non-volatile compositions as well as consumer perceptions in sensory evaluation, is crucial to better understand the impact of these compounds.

## CRediT authorship contribution statement

**Jiaqiang Luo:** Writing – review & editing, Writing – original draft, Visualization, Validation, Methodology, Investigation, Formal analysis, Data curation, Conceptualization. **Damian Frank:** Writing – review & editing, Writing – original draft, Supervision, Methodology, Funding acquisition. **Jayashree Arcot:** Writing – review & editing, Supervision, Conceptualization.

## Declaration of competing interest

The authors declare that they have no known competing financial interests or personal relationships that could have appeared to influence the work reported in this paper.

## Data Availability

Data including all GC–O data will be made available on request.

## Appendix A Supplementary data

Supplementary material 1: Origins and ingredients of investigated PBSAs. Image 1

Supplementary material 2: Nutritional information of seafood and plant-based seafood. Image 2

Supplementary material 3: Supplementary Tables. Image 3
