# Supplementary material for: Creating alternative seafood flavour from non-animal ingredients: A review of key flavour molecules relevant to seafood
Source: Food Chem X. 2024 Apr 24;22:101400. doi: 10.1016/j.fochx.2024.101400 (PMC11088277; doi:10.1016/j.fochx.2024.101400)
Supplement: Supplementary file 3 — Supplementary material 3: Supplementary Tables. [file mmc3.docx]

# SUPPLEMENTARY MATERIALS

**Authors:** Jiaqiang Luo^a,*^, Damian Frank^b^, Jayashree Arcot^a^

**Affiliations:** ^a^Food and Health, School of Chemical Engineering, Faculty of Engineering, UNSW Sydney, Kensington, NSW 2052, Australia, ^b^All G Foods, Waterloo, NSW 2017, Australia

**Corresponding author’s email address:** Email: xavier.luo@unsw.edu.au

# Description

This present data provides supplementary information to our study. Odour-active volatile compounds identified in crustacean, molluscs, and finfish are summarised in **Supplementary tables S1**, **S2**, and **S3**, respectively. The article comprises two data files: The first file summarises ingredients of the investigated plant-based seafood products. The second file contains nutritional information of seafood sourced from the USDA and FSANZ databases, along with nutritional data for 41 commercially available plant-based seafood products, extracted from their nutrition labels.

**Supplementary Table S1.** Odour-active volatile compounds identified in crustaceans.

| **Compound** | **Odour descriptor^** | **Class*** | **^1^Crab** | **^2^Lobster** | **^3^Prawn** |
| --- | --- | --- | --- | --- | --- |
| acetic acid | acidic, onion, pungent, sour, vinegar | acid |  | 1 | 2 |
| 2-methylpropanoic acid | fruity, sweat | acid |  | 1 | 1 |
| butanoic acid | cheese, sweat | acid |  | 1 | 1 |
| 3-methylbutanoic acid | burnt, cheese, metallic, sour, sweat | acid |  | 1 | 1 |
| 2-methylbutanoic acid | fruity, sweat | acid |  | 1 | 2 |
| pentanoic acid | cheese, fruity, sweaty | acid |  | 1 | 1 |
| *(E)*-3-heptanoic acid | sour, faecal, mouldy | acid |  | 1 |  |
| benzeneacetic acid | beewax, honey, sweet | acid |  | 1 | 1 |
| decanoic acid | leather, musty | acid |  | 1 | 1 |
| dimethylamine | fishy, ammonia | amine | 5 |  |  |
| trimethylamine | amine, ammonia, crustacean-like, fish-like | amine |  | 2 |  |
| 1-penten-3-ol | cabbage, chemical, fish-like, floral, grass, green, mushroom, paint, plastic | alcohol | 3 |  |  |
| 3‐methyl‐1‐butanol | bitter, chocolate | alcohol |  | 1 |  |
| 1-pentanol | earthy, floral, fruity, mushroom | alcohol | 2 |  |  |
| 1-octen-3-ol | earthy, fatty, fermented, fish-like, fruity, grass, mushroom, potato | alcohol | 3 |  | 1 |
| 1-octanol | chemical, cucumber, green metallic, roasted | alcohol | 1 |  |  |
| 3-decanol | green, grass | alcohol | 1 |  |  |
| 2-methylpropanal | burnt, chocolate, malt, nutty | aldehyde | 1 |  |  |
| 3-methylbutanal | caramel, chocolate, fish-like, grass, malt, sweet, vegetable | aldehyde | 6 | 1 |  |
| 2-methylbutanal | chocolate, malt, mushroom, nutty, sweet | aldehyde | 3 | 1 |  |
| pentanal | grass, green | aldehyde | 2 |  |  |
| hexanal | burnt, earthy, fish-like, floral, fresh, fruity, garlic, grass, green, insect, leafy, nutty, plastic | aldehyde | 6 | 2 |  |
| furfural | cheese-like | aldehyde | 1 |  |  |
| *(E)*-2-hexenal | eucalyptus, fish-like, fruity, grass, green, moss, mushroom | aldehyde | 1 |  |  |
| heptanal | chocolate, citrus, earthy, fatty, floral, fruity, grass, green, mushroom, nutty, potato | aldehyde | 4 | 1 |  |
| *(Z)*-4-heptenal | biscuit, cooked, creamy, crustacean-like, fatty, fish-like, grass, leafy, marine, potato, rancid, vegetable | aldehyde | 5 | 2 | 1 |
| *(E)*-2-heptenal | fatty, fish-like, grass, roasted, sulphur | aldehyde | 1 |  |  |
| benzaldehyde | burnt, floral, fruity, green, metallic, nutty, paint, roasted, sweet, woody | aldehyde | 7 |  |  |
| octanal | citrus, fatty, fish-like, floral, fruity, grass, green, leafy, nutty, oily, orange, potato, sweet, waxy, wine | aldehyde | 2 |  |  |
| benzeneacetaldehyde | floral, fruity, green, honey, moss, spicy, sweet | aldehyde | 2 |  | 1 |
| *(Z,Z)*-3,6-nonadienal | fatty, green, melon | aldehyde |  | 1 |  |
| nonanal | citrus, corn, cucumber, fatty, fish-like, floral, fruity, geranium, grass, green, hospital, marine, meaty, oily, plastic, vegetable | aldehyde | 5 |  |  |
| 2-n-butylacrolein | floral, sweet | aldehyde | 2 |  |  |
| *(Z)*-2-nonenal | cardboard, fatty, green | aldehyde |  | 1 |  |
| *(E,Z)*-2,6-nonadienal | cucumber, fatty, floral, grass, green, insect, melon | aldehyde |  | 2 | 1 |
| *(E)*-2-nonenal | bitter, cardboard, cucumber, earthy, fatty, fish-like, floral, green, hay, moss, musty, nutty, rotten, stale, woody | aldehyde |  |  | 1 |
| 4-ethylbenzaldehyde | anise, fruity, green, marine, minty, nutty | aldehyde | 2 |  |  |
| *(Z)*-4-decenal | vegetable | aldehyde | 2 |  |  |
| *(E)*-4-decenal | potato | aldehyde | 1 |  |  |
| *(E,E)*-2,4-nonadienal | fatty, fish-like, green, melon, putrid | aldehyde |  | 1 | 1 |
| decanal | cardboard, citrus, cucumber, fish-like, floral, fresh, grass, green, marine, oily, plastic | aldehyde | 2 |  |  |
| (*E*,*Z*,*E*)-2,4,6-nonatrienal | fatty, oat | aldehyde |  | 1 | 1 |
| *(E,E)*-2,4-decadienal | chemical, cooked, cucumber, fatty, fried, grass, green | aldehyde |  | 2 | 1 |
| *(E,Z)*-2,4-decadienal | fatty, fish-like, green, medicinal, plastic, popcorn, roasted | aldehyde |  |  | 1 |
| vanillin | coconut, vanilla | aldehyde |  | 1 | 1 |
| *(E)*-4,5-epoxy-*(E)*-2-decenal | metallic | aldehyde |  | 1 | 1 |
| 2,4,6-tribromoanisole | marine, sea breeze-like | anisole |  |  | 1 |
| toluene | dusty, floral, grass, paint | benzene | 4 |  |  |
| ethylbenzene | floral, nutty | benzene | 1 |  |  |
| p-cresol | faecal, horse stable, stable | benzene |  |  | 1 |
| 2-methy-furan | mushroom-like | furan | 1 |  |  |
| 2-ethyl-furan | corn, roasted, sweet | furan | 1 |  |  |
| 2-butyl-furan | green, sweet | furan | 1 |  |  |
| 2-pentyl-furan | fish-like, grass, green, sweet | furan | 4 |  |  |
| 2-(2-pentenyl)furan | tomato | furan | 2 |  |  |
| furaneol | caramel, strawberry, sweet | furan |  | 2 | 1 |
| indole | faecal, hay, mothball, musty, woody | indole |  | 1 | 2 |
| 3-methylindole | chlorine, faecal, mothball, pungent | indole |  | 1 | 1 |
| 2‐butanone | cheese, chemical, plastic, roasted | ketone | 3 |  |  |
| 2,3-butanedione | butter, caramel, creamy, rotten, sour, sweet | ketone | 2 | 2 |  |
| 1-penten-3-one | chemical, green, paint | ketone | 1 |  |  |
| 2,3-pentanedione | butter, caramel, fruity, grass, malt, meaty, milk, rotten, sweet | ketone | 1 |  |  |
| acetoin | floral, dusty, buttery | ketone | 1 |  |  |
| 1-hexen-3-one | plastic | ketone |  | 1 |  |
| 2-heptanone | bitter, cheese, grass, meaty, mouldy | ketone | 4 | 1 |  |
| 1-octen-3-one | earthy, egg, green, mushroom, sweet | ketone | 2 | 2 | 1 |
| *(Z)*-1,5-octadien-3-one | geranium, metallic, mushroom, plant | ketone |  | 2 | 1 |
| 6‐methyl‐5‐hepten‐2‐one | alcohol, chemical, citrus, floral, green, sourplum, spicy, vegetable | ketone | 3 |  |  |
| acetophenone | animal, grass, nutty | ketone | 1 |  |  |
| *(E,E)*-3,5-octadien-2-one | chemical, milk, plastic, roasted, rosewater, sweet, tomato | ketone | 3 |  |  |
| 2-nonanone | blue cheese, chocolate, fruity | ketone | 4 |  |  |
| sotolone | seasoning-like | ketone |  | 1 | 4 |
| 2-decanone | fruity, mushroom, musty, orange, sweat, tomato | ketone | 2 |  |  |
| abhexone | seasoning | ketone |  |  | 1 |
| 3-methyl-2,4-nonanedione | anise, fish-like, fruity, green, hay sweet | ketone |  | 1 | 1 |
| 6-undecanone | grass | ketone |  |  | 1 |
| 2-aminoacetophenone | grape, rubber | ketone |  | 1 | 1 |
| γ-decalactone | coconut, peach, sweet | lactone |  | 1 | 1 |
| pyrazine | dusty, nutty | pyrazine | 3 |  |  |
| 2-methylpyrazine | corn, nutty, popcorn, rubber, sweet | pyrazine | 4 |  |  |
| 2,5-dimethylpyrazine | burnt, chocolate, corn, medicinal, milk, nutty, plastic, popcorn, roasted, sweet | pyrazine | 4 |  | 1 |
| 2,6-dimethylpyrazine | nutty | pyrazine |  |  | 1 |
| 2,3-dimethylpyrazine | butter, green, nutty, popcorn | pyrazine | 2 |  | 1 |
| 2,3,5-trimethylpyrazine | broth, burnt, chocolate, coffee, earthy, nutty, popcorn, roasted | pyrazine | 2 | 2 | 1 |
| 2-acetylpyrazine | burnt, grilled, metallic, nutty, popcorn, potato, roasted | pyrazine |  | 1 | 1 |
| 2-ethyl-3,5-dimethylpyrazine | earthy, metallic, mouldy, nutty, plastic, roasted | pyrazine |  | 1 | 1 |
| 2-acetyl-3-methylpyrazine | burnt, nutty, popcorn | pyrazine | 1 | 1 |  |
| 3-ethyl-2,5-dimethylpyrazine | chocolate, musty | pyrazine | 1 |  |  |
| 2-methylpyridine | fish-like | pyridine | 2 |  |  |
| 3-methylpyridine | fish-like, green | pyridine | 2 |  |  |
| 2-ethylpyridine | potato mud | pyridine | 2 |  |  |
| 2-acetylpyridine | popcorn-like | pyridine |  | 1 | 1 |
| 3-propylpyridine | earthy | pyridine | 1 |  |  |
| 2-acetyl-1-pyrroline | nutty, oily, popcorn, roasted | pyrrole | 3 | 3 | 1 |
| dimethyl disulphide | onion, sulphur | sulphide | 1 |  |  |
| dimethyl trisulphide | cabbage, fermented, garlic, green, marine, metallic, putrid, rotten, savoury, sulphur, vegetable, woody | sulphide | 1 | 1 |  |
| linalool | citrus, floral, green | terpene |  |  | 1 |
| β-damascenone | apple, tea | terpene |  | 1 |  |
| geosmin | earthy, musty | terpene |  |  | 1 |
| α-cadinol | hay, woody | terpene |  |  | 1 |
| methanethiol | meaty, putrid, rotten, sulphur | thiol |  | 3 |  |
| 2-methylthiophene | onion, sulphury | thiol | 1 |  |  |
| 2-methyl-3-furanthiol | meaty, vitamin | thiol |  | 1 |  |
| 2-furfurylthiol | coffee, roasted | thiol |  | 1 |  |
| methional | chemical, meaty, potato, roasted, salty, soy sauce | thiol | 3 |  | 2 |
| 3-thiophanone | grassy, garlic like | thiol |  |  | 1 |
| 2,4,5-trimethylthiazole | metallic, sulphury | thiol |  | 1 |  |
| 3-formylthiophene | sulphury | thiol | 1 |  |  |
| 2-acetylthiazole | burnt, earthy, grilled, meaty, milk, nutty, popcorn, roasted, sulphur, sweet | thiol | 3 | 2 | 1 |
| 2-acetyl-2-thiazoline | grilled, nutty, popcorn, roasted | thiol |  |  | 2 |
| 3-methyl-2-thiophenecarboxaldehyde | chocolate | thiol | 1 |  |  |
| benzothiofuran | mothball | thiol |  | 1 |  |
| benzothiazole | fruity, green, leather, phenolic, plastic, roasted, rubber, seaweed | thiol |  | 1 | 1 |
| *(Z)*-3-methyl-1-butene-1-thiol | roasted, sulphur | thiol |  |  | 1 |
| pyrrolidine | chemical, egg, rancid | other | 3 |  |  |

Notes: The numbers indicate the quantity of studies that have confirmed the presence of the compound. ^ Typical odour descriptors were primarily adopted from the study by Jones, Rocker [1], with missing odour information supplemented by findings from other seafood studies. *Compound classes include derivatives. ^1^Compounds in crabs were identified in the following studies: [2-10]. ^2^Compounds in lobsters: [11-13]. ^3^Compounds in prawns: [13, 14].

**Supplementary Table S2.** Odour-active volatile compounds identified in molluscs.

| **Compound** | **Odour descriptor^** | **Class** | **^1^Clam** | **^2^Mussel** | **^3^Oyster** | **^4^Scallop** | **^5^Squid** |
| --- | --- | --- | --- | --- | --- | --- | --- |
| butanoic acid | cheese, sweat | acid |  |  |  |  | 1 |
| 3-methylbutanoic acid | burnt, cheese, metallic, sour, sweat | acid |  |  |  |  | 1 |
| octanoic acid | fresh, moss | acid |  |  | 1 |  |  |
| trimethylamine | amine, ammonia, crustacean-like, fish-like | amine |  |  |  | 1 |  |
| 1-propanol | chemical, fruity, plastic | alcohol |  | 2 |  |  |  |
| 1-penten-3-ol | cabbage, chemical, fish-like, floral, grass, green, mushroom, paint, plastic | alcohol |  |  |  | 1 |  |
| *(E)*-2-penten-1-ol | butter, fish-like, green, marine, metallic, mushroom | alcohol |  | 2 | 2 |  |  |
| *(E)*-3-hexen-1-ol | fresh, green, moss | alcohol |  |  | 1 |  |  |
| *(Z)*-3-hexen-1-ol | green, marine, woody | alcohol |  | 1 |  |  |  |
| 1‐hexanol | alcohol, fish-like, green | alcohol | 1 |  |  |  |  |
| 1‐heptanol | fermented, potato, savoury | alcohol | 1 |  |  |  |  |
| 1-octen-3-ol | earthy, fatty, fermented, fish-like, fruity, grass, mushroom, potato | alcohol | 1 |  | 2 | 1 |  |
| 3-octanol | moss, sulphur | alcohol |  |  | 2 |  |  |
| 2-ethyl-1-hexanol | cucumber, earthy, fish-like, grass, green, mushroom, roasted, vegetable | alcohol | 1 |  |  |  |  |
| 1-octanol | chemical, cucumber, green metallic, roasted | alcohol | 1 |  | 2 |  |  |
| 2-nonanol | chemical, citrus, cucumber, fresh, fruity, moss, plastic | alcohol |  | 1 | 2 |  |  |
| *(E,Z)*-3,6-nonadien-1-ol | cucumber, marine | alcohol |  |  | 2 |  |  |
| 1-dodecanol | pungent, vegetable | alcohol | 1 |  |  |  |  |
| 2-methylpropanal | burnt, chocolate, malt, nutty | aldehyde |  |  |  | 1 |  |
| 2-methyl-1-butanal | earthy-musty | aldehyde |  |  |  | 1 |  |
| *(E)*‐2‐pentenal | fish-like, grass, green, marine, plant | aldehyde |  |  | 2 |  |  |
| hexanal | burnt, earthy, fish-like, floral, fresh, fruity, garlic, grass, green, insect, leafy, nutty, plastic | aldehyde | 1 | 2 | 1 | 1 |  |
| *(E)*-2-hexenal | eucalyptus, fish-like, fruity, grass, green, moss, mushroom | aldehyde | 1 |  |  |  |  |
| heptanal | chocolate, citrus, earthy, fatty, floral, fruity, grass, green, mushroom, nutty, potato | aldehyde | 1 | 1 |  |  |  |
| *(Z)*-4-heptenal | biscuit, cooked, creamy, crustacean-like, fatty, fish-like, grass, leafy, marine, potato, rancid, vegetable | aldehyde |  | 2 | 2 |  |  |
| *(E)*-2-heptenal | f fatty, fish-like, grass, roasted, sulphur | aldehyde |  | 2 |  |  |  |
| octanal | citrus, fatty, fish-like, floral, fruity, grass, green, leafy, nutty, oily, orange, potato, sweet, waxy, wine | aldehyde | 1 | 2 | 2 | 1 |  |
| *(E,E)*-2,4-heptadienal | aromatic, cooked, cucumber, fatty, fish-like, grass, green, hay, insect, marine, moss, mushroom, oil, potato, roasted, vegetable | aldehyde |  |  | 3 |  |  |
| *(Z)*-2-octenal | marine, mushroom | aldehyde |  |  | 1 |  |  |
| *(E)*-2-octenal | aromatic, citrus, cucumber, earthy, fatty, fish-like, fresh, lemon, moss, mouldy, nutty, oily, potato, pungent, roasted, savoury, stale, toast | aldehyde |  | 1 | 2 |  |  |
| nonanal | citrus, corn, cucumber, fatty, fish-like, floral, fruity, geranium, grass, green, hospital, marine, meaty, oily, plastic, vegetable | aldehyde | 1 |  |  | 1 |  |
| *(E,Z)*-2,6-nonadienal | cucumber, fatty, floral, grass, green, insect, melon | aldehyde |  |  | 3 |  |  |
| *(E)*-2-nonenal | bitter, cardboard, cucumber, earthy, fatty, fish-like, floral, green, hay, moss, musty, nutty, rotten, stale, woody | aldehyde |  | 1 |  |  |  |
| 4-ethylbenzaldehyde | anise, fruity, green, marine, minty, nutty | aldehyde |  | 1 | 1 |  |  |
| decanal | cardboard, citrus, cucumber, fish-like, floral, fresh, grass, green, marine, oily, plastic | aldehyde | 1 |  | 3 | 1 |  |
| toluene | dusty, floral, grass, paint | benzenoid |  |  |  | 1 |  |
| p-xylene | chemical, phenolic | benzenoid |  |  |  | 1 |  |
| m-xylene | phenolic, plastic | benzenoid |  | 1 |  |  |  |
| o-xylene | chemical, plastic, vegetable | benzenoid |  | 1 |  |  |  |
| styrene | balsamic, gasoline | benzenoid | 1 |  |  |  |  |
| 1,2,4-trimethyl-benzene | plastic | benzenoid |  | 1 |  |  |  |
| benzyl alcohol | floral, herbaceous, nutty, woody | benzenoid | 1 |  |  |  |  |
| eugenol | lilac | benzenoid | 1 |  |  |  |  |
| 2-ethyl-furan | corn, roasted, sweet | furan |  |  |  | 1 |  |
| 2-pentyl-furan | fish-like, grass, green, sweet | furan |  |  |  | 1 |  |
| decane | fishy | hydrocarbon |  |  |  | 1 |  |
| furaneol | caramel, strawberry, sweet | furan | 1 |  |  |  | 1 |
| 2,3-butanedione | butter, caramel, creamy, rotten, sour, sweet | ketone |  | 2 |  |  |  |
| 1-penten-3-one | chemical, green, paint | ketone |  |  | 1 |  |  |
| 2,3-pentanedione | butter, caramel, fruity, grass, malt, meaty, milk, rotten, sweet | ketone |  |  | 1 |  |  |
| *(E,E,Z)*-1,3,5-octatriene | cheese, green, plastic | ketone |  |  | 2 |  |  |
| 2-heptanone | bitter, cheese, grass, meaty, mouldy | ketone |  |  |  | 1 |  |
| 1-octen-3-one | earthy, egg, green, mushroom, sweet | ketone |  |  | 1 |  |  |
| 3-octanone | earthy-musty | ketone |  |  |  | 1 |  |
| 6‐methyl‐5‐hepten‐2‐one | alcohol, chemical, citrus, floral, green, sourplum, spicy, vegetable | ketone |  |  | 1 |  |  |
| acetophenone | animal, grass, nutty | ketone |  |  | 1 |  |  |
| sotolone | seasoning-like | ketone |  |  |  |  | 1 |
| 2-undecanone | cucumber, floral, fresh, fruity, green, nutty, peach, sweet | ketone |  |  | 2 |  |  |
| 1-hydroxy-2-acetone | stench | ketone |  |  |  | 1 |  |
| 2-methylpyrazine | corn, nutty, popcorn, rubber, sweet | pyrazine | 1 |  |  |  |  |
| 2,5-dimethylpyrazine | burnt, chocolate, corn, medicinal, milk, nutty, plastic, popcorn, roasted, sweet | pyrazine | 1 |  |  | 1 |  |
| 2,6-dimethylpyrazine | nutty | pyrazine | 1 |  |  |  |  |
| 2-ethylpyrazine | grilled, nutty | pyrazine |  | 2 | 2 |  |  |
| 2,3-dimethylpyrazine | butter, green, nutty, popcorn | pyrazine | 1 |  |  |  |  |
| 2,3,5-trimethylpyrazine | broth, burnt, chocolate, coffee, earthy, nutty, popcorn, roasted | pyrazine |  |  |  | 1 |  |
| 1-acetylpyrazine | burnt, grilled, metallic, nutty, popcorn, potato, roasted | pyrazine |  | 1 |  |  |  |
| 2-acetylpyrazine | burnt, grilled, metallic, nutty, popcorn, potato, roasted | pyrazine | 1 |  | 1 |  | 1 |
| 2-acetyl-3-methylpyrazine | burnt, nutty, popcorn | pyrazine | 1 |  |  |  |  |
| 2-ethylpyridine | potato mud | pyridine |  |  |  | 1 |  |
| 2-acetyl-1-pyrroline | nutty, oily, popcorn, roasted | pyrrole |  |  |  |  | 1 |
| dimethyl sulphide | cabbage, corn, crustacean-like, fish-like, green, marine, sulphur | sulphide |  | 2 | 1 | 1 |  |
| methyl allyl sulphide | sulphur | sulphide |  |  |  | 1 |  |
| diallyl sulphide | sulphur | sulphide |  |  |  | 1 |  |
| methyl allyl disulphide | sulphur, earthy-musty | sulphide |  |  |  | 1 |  |
| dimethyl trisulphide | cabbage, fermented, garlic, green, marine, metallic, putrid, rotten, savoury, sulphur, vegetable, woody | sulphide |  | 2 |  | 1 |  |
| diallyl disulphide | sulphur | sulphide |  |  |  | 1 |  |
| limonene | chemical, citrus, floral, fresh, green, moss, pine | terpene |  |  | 2 |  |  |
| methanethiol | meaty, putrid, rotten, sulphur | thiol |  |  |  | 1 |  |
| 1-propanethiol | sulphur | thiol |  |  |  | 1 |  |
| 2-methyl-3-furanthiol | meaty, vitamin | thiol |  |  |  |  | 1 |
| 2-furfurylthiol | coffee, roasted | thiol |  |  |  |  | 1 |
| methional | chemical, meaty, potato, roasted, salty, soy sauce | thiol | 2 | 2 | 1 |  | 1 |
| 4,5-dimethylthiazole | molt, sweat | thiol |  |  |  |  | 1 |
| 2-acetylthiazole | burnt, earthy, grilled, meaty, milk, nutty, popcorn, roasted, sulphur, sweet | thiol |  | 1 |  |  |  |
| 2-acetyl-2-thiazoline | grilled, nutty, popcorn, roasted | thiol | 1 | 1 |  | 1 |  |
| benzothiazole | fruity, green, leather, phenolic, plastic, roasted, rubber, seaweed | thiol | 1 |  |  |  |  |
| maltol | sweet | other | 1 |  |  |  |  |

Notes: The numbers indicate the quantity of studies that have confirmed the presence of the compound. ^ Typical odour descriptors were primarily adopted from the study by Jones, Rocker [1], with missing odour information supplemented by findings from other seafood studies. *Compound classes include derivatives. ^1^Compounds in clams were identified in the following studies: [15, 16]. ^2^Compounds in mussels: [17, 18]. ^3^Compounds in oysters: [19-21]. ^4^Compounds in scallops: [22]. ^5^Compounds in squids: [23].

**Supplementary Table S3.** Odour-active volatile compounds identified in finfish.

| **Compound** | **Odour descriptor** | **Class** | **^1^Bass** | **^2^Cod** | **3Hake** | **^4^Mullet** | **^5^Salmon** | **^6^Sardine** | **^7^Trout** | **^8^Turbot** | **^9^Yellowtail** |
| --- | --- | --- | --- | --- | --- | --- | --- | --- | --- | --- | --- |
| formic acid | sour, ammonia | acid |  |  |  |  |  |  |  |  |  |
| acetic acid | acidic, onion, pungent, sour, vinegar | acid |  |  |  |  |  |  | 1 |  |  |
| propanoic acid | sharp, acidic | acid |  |  |  |  |  |  | 1 |  |  |
| butanoic acid | cheese, sweat | acid |  |  |  |  |  |  | 1 |  |  |
| 3-methylbutanoic acid | burnt, cheese, metallic, sour, sweat | acid |  |  |  |  |  |  | 1 |  |  |
| 2-methylbutanoic acid | fruity, sweat | acid |  |  |  |  |  |  | 1 |  |  |
| pentanoic acid | cheese, fruity, sweaty | acid |  |  |  |  |  |  | 1 |  |  |
| hexanoic acid | musty, pungent | acid |  |  |  |  |  |  | 2 |  |  |
| octanoic acid | fresh, moss | acid |  |  |  |  |  |  | 1 |  |  |
| benzeneacetic acid | beewax, honey, sweet | acid |  |  |  |  |  |  | 1 |  |  |
| nonanoic acid | musty | acid |  |  |  |  |  |  | 2 |  |  |
| 4-ethyloctanoic acid | goat | acid |  |  |  |  |  |  | 1 |  |  |
| decanoic acid | leather, musty | acid |  |  |  |  |  |  | 1 |  |  |
| dodecanoic acid | rubbery, musty | acid |  |  |  |  |  |  | 1 |  |  |
| tetradecanoic acid | cheese, fatty, marine | acid |  |  |  |  | 1 |  |  |  |  |
| hexadecanoic acid | fatty, fresh, fruity, plastic | acid |  |  |  |  |  |  | 2 |  |  |
| oleic acid | alcohol, earthy, plastic | acid |  |  |  |  | 1 |  |  |  |  |
| trimethylamine | amine, ammonia, crustacean-like, fish-like | amine |  | 1 |  |  |  |  |  | 1 |  |
| 1-propanol | chemical, fruity, plastic | alcohol |  |  |  |  |  |  | 1 | 1 |  |
| 1-penten-3-ol | cabbage, chemical, fish-like, floral, grass, green, mushroom, paint, plastic | alcohol | 1 |  |  | 1 | 2 |  | 1 |  |  |
| *(E)*-2-penten-1-ol | butter, fish-like, green, marine, metallic, mushroom | alcohol | 1 |  |  |  |  | 1 | 1 | 1 |  |
| 4-methyl-2-pentanol | mushroom | alcohol |  |  |  |  |  |  |  | 1 |  |
| 1-pentanol | earthy, floral, fruity, mushroom | alcohol | 1 |  |  |  |  |  |  |  |  |
| *(Z)*-2-penten-1-ol | cabbage, cooked, mushroom, nutty | alcohol |  |  |  |  | 1 |  |  |  |  |
| 2-methyl-2-buten-1-ol | sweetness | alcohol |  |  |  |  |  |  | 1 |  |  |
| 3-penten-2-ol | oily, green | alcohol |  |  |  |  |  |  | 1 |  |  |
| 2-hexanol | green, sharp | alcohol |  |  |  |  |  |  | 1 |  |  |
| *(Z)*-3-hexen-1-ol | green, marine, woody | alcohol |  |  |  |  |  | 1 |  |  |  |
| 1‐hexanol | alcohol, fish-like, green | alcohol |  |  |  | 1 |  |  | 1 |  | 3 |
| *(E)*-2-hexen-1-ol | green, moss | alcohol |  |  |  |  |  |  | 1 | 1 |  |
| 1‐heptanol | fermented, potato, savoury | alcohol |  |  |  | 1 |  |  |  |  |  |
| *(Z,Z)*-1,5-octadien-3-ol | cooked, moss, mushroom | alcohol |  |  |  |  |  |  | 2 | 1 |  |
| 1-octen-3-ol | earthy, fatty, fermented, fish-like, fruity, grass, mushroom, potato | alcohol |  |  |  |  | 2 | 1 | 3 |  |  |
| 2-ethyl-1-hexanol | cucumber, earthy, fish-like, grass, green, mushroom, roasted, vegetable | alcohol |  |  |  |  | 1 | 1 | 2 |  |  |
| *(E)*-2-octen-1-ol | grass, green | alcohol |  |  |  |  | 1 |  | 1 |  |  |
| 1-octanol | chemical, cucumber, green metallic, roasted | alcohol |  |  |  |  |  |  | 1 |  |  |
| 1-nonen-3-ol | mushroom | alcohol | 1 |  |  |  |  |  |  |  |  |
| *(E)*-3-hepten-2-ol | cucumber, green | alcohol |  |  |  |  |  |  | 1 |  |  |
| 2-nonanol | chemical, citrus, cucumber, fresh, fruity, moss, plastic | alcohol |  |  |  |  |  | 1 | 1 | 1 |  |
| 1-nonanol | citrus, floral, green | alcohol |  |  |  |  |  |  | 1 |  |  |
| butoxyethoxyethanol | floral, minty, plastic | alcohol |  |  |  |  |  |  | 2 |  |  |
| decanol | fatty, plastic | alcohol |  |  |  |  | 1 |  |  |  |  |
| 1-dodecanol | pungent, vegetable | alcohol |  |  |  |  |  |  | 1 |  |  |
| 1-hexadecanol | fruity, flowery | alcohol |  |  |  |  |  |  | 1 |  |  |
| 2-(dodecyloxy)ethanol | cooked, green | alcohol |  |  |  |  |  |  | 1 |  |  |
| acetaldehyde | alcohol, chemical, sweet | aldehyde |  | 1 |  |  | 1 |  | 1 |  |  |
| propanal | alcohol, fruity, green, sweet | aldehyde |  |  |  |  | 2 | 1 | 1 |  |  |
| 3-methylbutanal | caramel, chocolate, fish-like, grass, malt, sweet, vegetable | aldehyde |  | 2 |  |  | 1 |  |  |  |  |
| pentanal | grass, green | aldehyde |  |  |  |  | 1 |  |  |  |  |
| *(E)*‐2‐pentenal | fish-like, grass, green, marine, plant | aldehyde |  |  |  |  |  | 1 | 1 | 1 |  |
| *(Z)*-3-hexenal | grass, green, insect | aldehyde |  | 1 | 1 |  | 2 | 1 | 2 |  | 3 |
| hexanal | burnt, earthy, fish-like, floral, fresh, fruity, garlic, grass, green, insect, leafy, nutty, plastic | aldehyde | 2 | 1 |  |  | 3 | 2 | 3 | 2 | 3 |
| *(E)*-2-hexenal | eucalyptus, fish-like, fruity, grass, green, moss, mushroom | aldehyde |  |  |  | 2 | 1 | 1 | 1 | 1 |  |
| heptanal | chocolate, citrus, earthy, fatty, floral, fruity, grass, green, mushroom, nutty, potato | aldehyde | 1 | 1 |  | 1 | 2 | 2 | 2 | 1 |  |
| *(Z)*-4-heptenal | biscuit, cooked, creamy, crustacean-like, fatty, fish-like, grass, leafy, marine, potato, rancid, vegetable | aldehyde | 1 | 1 | 1 | 1 | 2 | 2 | 3 | 2 | 3 |
| *(E)*-2-heptenal | fatty, fish-like, grass, roasted, sulphur | aldehyde |  |  |  | 1 | 1 | 1 | 1 | 1 |  |
| benzaldehyde | burnt, floral, fruity, green, metallic, nutty, paint, roasted, sweet, woody | aldehyde |  |  |  | 1 | 2 |  | 1 | 1 |  |
| octanal | citrus, fatty, fish-like, floral, fruity, grass, green, leafy, nutty, oily, orange, potato, sweet, waxy, wine | aldehyde | 1 |  |  | 1 | 2 | 2 | 4 | 1 |  |
| benzeneacetaldehyde | floral, fruity, green, honey, moss, spicy, sweet | aldehyde |  |  |  |  | 2 |  |  |  |  |
| *(E)*-2-octenal | aromatic, citrus, cucumber, earthy, fatty, fish-like, fresh, lemon, moss, mouldy, nutty, oily, potato, pungent, roasted, savoury, stale, toast | aldehyde |  |  |  | 1 | 2 | 3 |  |  |  |
| *(Z,Z)*-3,6-nonadienal | fatty, green, melon | aldehyde |  |  |  |  | 1 |  | 1 |  |  |
| nonanal | citrus, corn, cucumber, fatty, fish-like, floral, fruity, geranium, grass, green, hospital, marine, meaty, oily, plastic, vegetable | aldehyde | 2 |  |  | 1 | 2 |  | 3 |  |  |
| *(E,E)*-2,4-octadienal | cooked, cucumber, fatty, floral, green, meaty, phenolic, pine, roasted, rosewater, sweet | aldehyde |  |  |  | 1 | 2 | 1 | 1 |  |  |
| *(Z)*-2-nonenal | cardboard, fatty, green | aldehyde |  |  | 1 |  |  |  | 1 |  |  |
| *(E,E)*-2,6-nonadienal | cucumber | aldehyde |  |  |  |  |  |  | 1 |  | 2 |
| *(E,Z)*-2,6-nonadienal | cucumber, fatty, floral, grass, green, insect, melon | aldehyde | 1 | 1 | 1 |  | 3 | 2 | 3 | 2 | 1 |
| *(E)*-2-nonenal | bitter, cardboard, cucumber, earthy, fatty, fish-like, floral, green, hay, moss, musty, nutty, rotten, stale, woody | aldehyde |  |  |  | 1 | 3 |  | 2 | 2 |  |
| 4-ethylbenzaldehyde | anise, fruity, green, marine, minty, nutty | aldehyde |  |  |  |  |  |  | 1 | 1 |  |
| *(E,E)*-2,4-nonadienal | fatty, fish-like, green, melon, putrid | aldehyde |  | 1 |  |  | 2 |  |  |  |  |
| decanal | cardboard, citrus, cucumber, fish-like, floral, fresh, grass, green, marine, oily, plastic | aldehyde | 1 | 1 |  | 1 |  |  | 3 | 1 |  |
| *(E)*-2-decenal | cooked, fatty, plastic | aldehyde |  |  |  |  | 1 |  |  |  |  |
| (*E*,*Z*,*E*)-2,4,6-nonatrienal | fatty, oat | aldehyde |  |  | 1 |  |  |  |  |  |  |
| *(E,E)*-2,4-decadienal | chemical, cooked, cucumber, fatty, fried, grass, green | aldehyde |  |  |  | 1 | 1 | 1 | 1 |  |  |
| undecanal | anise, fruity, herbaceous, minty, sweet | aldehyde |  | 2 | 1 |  | 3 |  | 2 | 1 |  |
| *(E,Z)*-2,4-decadienal | fatty, fish-like, green, medicinal, plastic, popcorn, roasted | aldehyde |  |  | 1 |  | 1 |  | 1 |  |  |
| *(E)*- 2-undecenal | oily, plastic | aldehyde |  |  |  | 1 |  |  |  |  |  |
| vanillin | coconut, vanilla | aldehyde |  |  |  |  |  |  | 1 |  |  |
| *(E)*-4,5-epoxy-*(E)*-2-decenal | metallic | aldehyde |  | 1 |  |  |  |  | 1 |  |  |
| *(E,Z,Z)*-2,4,7-tridecatrienal | blood | aldehyde |  |  |  |  |  |  | 1 |  |  |
| tetradecanal | marine, plastic, woody | aldehyde |  |  |  |  | 1 |  |  |  |  |
| hexadecanal | chemical, fatty, marine | aldehyde |  |  |  | 1 | 1 |  |  |  |  |
| *(Z)*-13-octadecenal | fatty | aldehyde |  |  |  |  |  |  |  |  |  |
| 4-ethylvanillin | honey, sweet | aldehyde |  |  |  |  |  |  | 1 |  |  |
| 2,4,6-trichloroanisole | cork | anisole |  |  |  |  |  |  | 1 |  |  |
| 2,3,4-trichloroanisole | medicinal | anisole |  |  |  |  |  |  | 1 |  |  |
| toluene | dusty, floral, grass, paint | benzenoid | 1 |  |  |  | 1 |  |  |  |  |
| p-xylene | chemical, phenolic | benzenoid |  |  |  |  | 1 |  |  |  |  |
| m-xylene | phenolic, plastic | benzenoid |  |  |  |  | 1 |  |  |  |  |
| o-xylene | chemical, plastic, vegetable | benzenoid |  |  |  |  | 2 |  |  |  |  |
| benzyl alcohol | floral, herbaceous, nutty, woody | benzenoid |  |  |  |  | 1 |  |  | 1 |  |
| p-cresol | faecal, horse stable, stable | benzenoid |  |  |  |  |  |  | 1 |  |  |
| 2-methyldecalin | pungent, animal | benzenoid |  |  |  |  |  |  | 1 |  |  |
| 2-phenoxyethanol | aromatic, bubble gum, chemical, minty | benzenoid |  |  |  | 1 |  |  | 2 |  |  |
| 1,2-dimethyldecahydronaphthalene | cucumber, garlic | benzenoid |  |  |  |  |  |  | 1 |  |  |
| ethyl butanoate | sweet, vomit | ester |  | 1 |  |  |  |  |  |  |  |
| benzyl acetate | floral, green | ester |  |  |  |  |  |  | 1 |  |  |
| ethyl decanoate | waxy, fruity, sweet | ester |  |  |  |  | 1 |  |  |  |  |
| ethyl hexadecanoate | fatty, floral, fruity, green, hairspary | ester |  |  |  |  |  |  | 2 |  |  |
| 2-ethyl-furan | corn, roasted, sweet | furan |  |  |  |  | 1 |  |  |  |  |
| 2-pentyl-furan | fish-like, grass, green, sweet | furan |  |  |  |  | 1 |  |  |  |  |
| 2-(2-pentenyl)furan | tomato | furan |  |  |  |  |  |  |  | 1 |  |
| 2-pentene | gas, chemical-like | hydrocarbon |  |  |  |  |  | 1 |  |  |  |
| menthatriene | cucumber, floral, green | hydrocarbon |  |  |  |  | 1 |  |  |  |  |
| tetradecane | cucumber | hydrocarbon | 1 |  |  |  |  |  |  |  |  |
| 1-pentadecene | plastic, rubber | hydrocarbon |  |  |  |  | 1 |  |  |  |  |
| hexadecane | fishy | hydrocarbon | 1 |  |  |  |  |  |  |  |  |
| 8-heptadecene | earthy, moss, plastic | hydrocarbon |  |  |  |  | 1 |  | 1 |  |  |
| heptadecane | cooking | hydrocarbon | 1 |  |  |  |  |  |  |  |  |
| pristane | fishy | hydrocarbon | 1 |  |  |  |  |  |  |  |  |
| *(E)*-3-octadecene | cheese, plastic | hydrocarbon |  |  |  |  | 1 |  |  |  |  |
| phytane | fishy | hydrocarbon | 1 |  |  |  |  |  |  |  |  |
| nonadecane | alkane | hydrocarbon | 1 |  |  |  |  |  |  |  |  |
| eicosane | alkane | hydrocarbon | 1 |  |  |  |  |  |  |  |  |
| furaneol | caramel, strawberry, sweet | furan |  |  |  |  |  |  | 1 |  |  |
| indole | faecal, hay, mothball, musty, woody | indole |  |  |  |  |  |  | 1 |  |  |
| skatole | faecal | indole |  |  |  |  |  |  | 1 |  |  |
| 2‐butanone | cheese, chemical, plastic, roasted | ketone |  |  |  |  |  | 1 |  |  |  |
| 2,3-butanedione | butter, caramel, creamy, rotten, sour, sweet | ketone | 1 | 1 |  |  | 2 | 2 | 2 | 2 | 3 |
| 1-penten-3-one | chemical, green, paint | ketone |  |  |  |  | 1 | 1 |  |  | 3 |
| 2,3-pentanedione | butter, caramel, fruity, grass, malt, meaty, milk, rotten, sweet | ketone | 1 | 1 |  | 1 | 2 | 3 | 1 |  | 3 |
| 3-pentanone | caramel, sweet | ketone |  | 1 |  |  |  |  | 1 |  |  |
| acetoin | floral, dusty, buttery | ketone |  |  |  | 1 | 1 |  | 2 |  |  |
| *(E,E,Z)*-1,3,5-octatriene | cheese, green, plastic | ketone |  |  |  |  |  | 1 |  | 1 |  |
| 2-heptanone | bitter, cheese, grass, meaty, mouldy | ketone |  |  |  |  |  |  |  | 1 |  |
| 1-octen-3-one | earthy, egg, green, mushroom, sweet | ketone | 1 | 2 | 1 |  | 2 | 2 | 2 |  | 2 |
| 2,3‐octanedione | cooked, savoury | ketone |  |  |  | 1 |  |  |  |  |  |
| *(Z)*-1,5-octadien-3-one | geranium, metallic, mushroom, plant | ketone |  | 2 | 1 |  | 1 | 1 | 2 |  |  |
| 6‐methyl‐5‐hepten‐2‐one | alcohol, chemical, citrus, floral, green, sourplum, spicy, vegetable | ketone |  | 1 |  |  |  |  | 1 |  |  |
| 3-octen-2-one | fatty, spicy | ketone |  |  |  |  |  | 1 |  |  |  |
| *(E,E)*-3,5-octadien-2-one | chemical, milk, plastic, roasted, rosewater, sweet, tomato | ketone |  |  |  |  | 1 |  |  |  |  |
| 2-nonanone | blue cheese, chocolate, fruity | ketone |  |  |  |  |  | 1 |  |  |  |
| *(E,Z)*-3,5-octadien-2-one | cucumber, fatty, floral, fruity, green | ketone |  |  | 1 |  |  | 1 |  |  |  |
| sotolone | seasoning-like | ketone |  |  |  |  |  |  | 1 |  |  |
| 2-decanone | fruity, mushroom, musty, orange, sweat, tomato | ketone |  |  |  |  |  |  | 1 |  |  |
| 3-methyl-2,4-nonanedione | anise, fish-like, fruity, green, hay sweet | ketone |  |  | 1 |  |  |  |  |  |  |
| 2-undecanone | cucumber, floral, fresh, fruity, green, nutty, peach, sweet | ketone |  |  |  |  | 1 |  | 1 | 2 |  |
| 2-pentadecanone | plastic, burnt | ketone |  |  |  | 1 |  |  |  |  |  |
| pristane | green, cooked | ketone |  |  |  |  |  |  | 1 |  |  |
| γ-crotonolactone | butter, cooked | lactone |  |  |  | 1 |  |  |  |  |  |
| γ-butyrolactone | oily | lactone |  |  |  | 1 |  |  | 1 |  |  |
| γ-nonalactone | coconut, sweet | lactone |  |  |  |  |  |  | 1 |  |  |
| γ-decalactone | coconut, peach, sweet | lactone |  |  |  |  |  |  | 1 |  |  |
| coumarin | grass, green | lactone |  |  |  |  |  |  | 1 |  |  |
| γ-dodecalactone | fruity, peach-like | lactone |  |  |  |  |  |  | 1 |  |  |
| pantolactone | burnt, chemical | lactone |  |  |  | 1 |  |  | 1 |  |  |
| γ-*(Z)*-6-dodecenolactone | peach-like, fresh | lactone |  |  |  |  |  |  | 1 |  |  |
| 2,5-dimethylpyrazine | burnt, chocolate, corn, medicinal, milk, nutty, plastic, popcorn, roasted, sweet | pyrazine |  |  |  | 1 |  |  |  |  |  |
| 2-ethylpyrazine | grilled, nutty | pyrazine |  |  |  |  |  |  | 1 |  |  |
| 2,3,5-trimethylpyrazine | broth, burnt, chocolate, coffee, earthy, nutty, popcorn, roasted | pyrazine |  |  |  |  |  |  | 1 |  |  |
| 1-acetylpyrazine | burnt, grilled, metallic, nutty, popcorn, potato, roasted | pyrazine |  |  |  |  |  |  |  | 1 |  |
| 2-ethyl-3,5-dimethylpyrazine | earthy, metallic, mouldy, nutty, plastic, roasted | pyrazine |  |  |  | 1 |  |  | 1 |  |  |
| 1-methyl-2-pyrrolidone | oily/fish oil | pyridine |  |  |  | 1 |  |  |  |  |  |
| 2-pyrrolidinone | popcorn | pyridine |  |  |  | 1 |  |  |  |  |  |
| 1-ethylpyrrole | chemical, roasted | pyrrole |  |  |  | 1 |  |  |  |  |  |
| 2-acetyl-1-pyrroline | nutty, oily, popcorn, roasted | pyrrole |  | 1 |  | 1 |  |  |  |  |  |
| dimethyl sulphide | cabbage, corn, crustacean-like, fish-like, green, marine, sulphur | sulphide |  | 1 |  |  |  | 1 |  |  |  |
| methyl allyl sulphide | sulphur | sulphide |  |  |  |  |  |  |  |  |  |
| dimethyl disulphide | onion, sulphur | sulphide |  | 1 |  |  | 1 |  |  |  |  |
| dimethyl trisulphide | cabbage, fermented, garlic, green, marine, metallic, putrid, rotten, savoury, sulphur, vegetable, woody | sulphide | 2 | 3 |  |  | 1 |  |  |  |  |
| dimethyl tetrasulphide | cabbage, putrid | sulphide |  | 2 |  |  |  |  |  |  |  |
| α-pinene | green, smoky | terpene |  |  |  |  |  |  | 1 |  |  |
| β‐pinene | pine, resin, turpentine | terpene |  |  |  |  |  |  |  |  | 1 |
| 3‐carene | eucalyptus, faecal, pungent | terpene |  |  |  |  |  |  | 1 |  |  |
| limonene | chemical, citrus, floral, fresh, green, moss, pine | terpene |  |  |  |  | 1 |  | 2 |  |  |
| β-ocimene | floral, herbal | terpene |  |  |  |  |  |  |  |  |  |
| γ-terpinene | green | terpene |  |  |  |  |  |  | 1 |  |  |
| linalool | citrus, floral, green | terpene |  |  |  | 1 |  |  |  |  |  |
| α-terpineol | earthy, eucalyptus, woody | terpene |  |  |  |  |  |  | 1 |  |  |
| 2-methylisoborneol | earthy, musty | terpene | 1 |  |  |  |  |  | 2 |  |  |
| β-caryophyllene | cucumber, earthy, green, moss, rotten | terpene |  |  |  |  |  |  | 2 |  |  |
| geosmin | earthy, musty | terpene | 1 |  |  |  | 1 |  | 2 |  |  |
| aromadendrene | cucumber, vanilla, floral | terpene |  |  |  |  | 1 |  |  |  |  |
| β-ionone | violet | terpene |  |  |  |  |  |  | 1 |  |  |
| rotundone | black pepper | terpene |  |  |  |  |  |  | 1 |  |  |
| farnesol | fruity | terpene |  |  |  |  | 1 |  |  |  |  |
| androstenone | sweat, urine | terpene |  |  |  |  |  |  | 1 |  |  |
| methanethiol | meaty, putrid, rotten, sulphur | thiol |  | 1 |  |  | 1 |  |  |  |  |
| methional | chemical, meaty, potato, roasted, salty, soy sauce | thiol | 1 | 2 |  | 1 | 3 | 3 | 3 | 1 | 3 |
| 2-formylthiophene | grilled, cooked | thiol |  |  |  |  | 1 |  | 1 |  |  |
| 2-acetylthiazole | burnt, earthy, grilled, meaty, milk, nutty, popcorn, roasted, sulphur, sweet | thiol |  |  |  |  |  | 1 |  |  |  |
| 3-isopropyl-2-methoxypyrazine | pea-like, green pepper | thiol |  |  |  |  |  |  | 1 |  |  |
| 2-acetyl-2-thiazoline | grilled, nutty, popcorn, roasted | thiol |  |  |  |  |  |  | 1 | 1 |  |
| 3-isobutyl-2-methoxypyrazine | pea-like, green pepper | thiol |  |  |  |  |  |  | 1 |  |  |
| benzothiazole | fruity, green, leather, phenolic, plastic, roasted, rubber, seaweed | thiol |  |  |  |  | 1 |  | 1 |  |  |

**Notes:** The numbers indicate the quantity of studies that have confirmed the presence of the compound. ^ Typical odour descriptors were primarily adopted from the study by Jones, Rocker [1], with missing odour information supplemented by findings from other seafood studies. *Compound classes include derivatives. ^1^Compounds in bass were identified in the following studies: [24-26]. ^2^Compounds in cods: [27-29]. ^3^Compounds in hakes: [30]. ^4^Compounds in mullets: [31, 32]. ^5^Compounds in salmons: [28, 33, 34]. ^6^Compounds in sardines: [35-37]. ^7^Compounds in trouts: [27, 38-41]. ^8^Compounds in turbots: [42, 43]. ^9^Compounds in yellowtails: [44-46].

# References

1. Jones, B.C., et al., *Systematic review of the odorous volatile compounds that contribute to flavour profiles of aquatic animals.* Reviews in Aquaculture, 2022. **14**(3): p. 1418-1477.

2. Chung, H.Y. and K.R. Cadwallader, *Aroma Extract Dilution Analysis of Blue Crab Claw Meat Volatiles.* Journal of Agricultural and Food Chemistry, 1994. **42**(12): p. 2867-2870.

3. Chung, H., F. Chen, and K. Cadwallader, *Cooked blue crab claw meat aroma compared with lump meat.* Journal of food science, 1995. **60**(2): p. 289-291.

4. Chen, D.W. and M. Zhang, *Determination of Odour-Active Compounds in the Cooked Meat of Chinese Mitten Crab (Eriocheir Sinensis) by Solid Phase Microextraction, Gas Chromatography-Olfactometry and Gas Chromatography-Mass Spectrometry.* Journal of Food and Drug Analysis, 2010. **18**(4): p. 290-296.

5. Yu, H.-Z. and S.-S. Chen, *Identification of characteristic aroma-active compounds in steamed mangrove crab (Scylla serrata).* Food research international, 2010. **43**(8): p. 2081-2086.

6. Gu, S.Q., et al., *Analysis of Key Odor Compounds in Steamed Chinese Mitten Crab (Eriocheir sinensis).* Advanced Materials Research, 2014. **941-944**: p. 1026-1035.

7. Wu, N., et al., *Characterization of Important Odorants in Steamed Male Chinese Mitten Crab (Eriocheir sinensis) using Gas Chromatography-Mass Spectrometry-Olfactometry.* Journal of Food Science, 2014. **79**(7): p. C1250-C1259.

8. Ji, S., et al., *Comparison of olfactometrically detected compounds and aroma properties of four different edible parts of Chinese mitten crab.* Fisheries Science, 2015. **81**: p. 1157-1167.

9. Wu, N. and X.C. Wang, *Comparison of Gender Differences in Nutritional Value and Key Odor Profile of Hepatopancreas of Chinese Mitten Crab (Eriocheir Sinensis).* Journal of Food Science, 2017. **82**(2): p. 536-544.

10. Wu, N. and X.-C. Wang, *Identification of important odorants derived from phosphatidylethanolamine species in steamed male Eriocheir sinensis hepatopancreas in model systems.* Food chemistry, 2019. **286**: p. 491-499.

11. Cadwallader, K.R., et al., *Evaluation of the aroma of cooked spiny lobster tail meat by aroma extract dilution analysis.* Journal of Agricultural and Food Chemistry, 1995. **43**(9): p. 2432-2437.

12. Lee, G.-H., O. Suriyaphan, and K. Cadwallader, *Aroma components of cooked tail meat of American lobster (Homarus americanus).* Journal of Agricultural and Food Chemistry, 2001. **49**(9): p. 4324-4332.

13. Mall, V. and P. Schieberle, *Characterization of key aroma compounds in raw and thermally processed prawns and thermally processed lobsters by application of aroma extract dilution analysis.* Journal of agricultural and food chemistry, 2016. **64**(33): p. 6433-6442.

14. Okabe, Y., et al., *Odor-active compounds contributing to the characteristic aroma of shrimp cooked whole, including shells and viscera.* European Food Research and Technology, 2019. **245**(1): p. 233-241.

15. Sekiwa, Y., K. Kubota, and A. Kobayashi, *Characteristic flavor components in the brew of cooked clam (Meretrix lusoria) and the effect of storage on flavor formation.* Journal of agricultural and food chemistry, 1997. **45**(3): p. 826-830.

16. Huang, X.-H., et al., *The effects of different extraction methods on the aroma fingerprint, recombination and visualization of clam soup.* Food & Function, 2021. **12**(4): p. 1626-1638.

17. Le Guen, S., C. Prost, and M. Demaimay, *Critical comparison of three olfactometric methods for the identification of the most potent odorants in cooked mussels (Mytilus edulis).* Journal of Agricultural and Food Chemistry, 2000. **48**(4): p. 1307-1314.

18. Le Guen, S., C. Prost, and M. Demaimay, *Evaluation of the representativeness of the odor of cooked mussel extracts and the relationship between sensory descriptors and potent odorants.* Journal of Agricultural and Food Chemistry, 2001. **49**(3): p. 1321-1327.

19. Piveteau, F., et al., *Aroma of fresh oysters Crassostrea gigas: composition and aroma notes.* Journal of Agricultural and Food Chemistry, 2000. **48**(10): p. 4851-4857.

20. Pennarun, A.L., C. Prost, and M. Demaimay, *Identification and origin of the character‐impact compounds of raw oyster Crassostrea gigas.* Journal of the Science of Food and Agriculture, 2002. **82**(14): p. 1652-1660.

21. Pennarun, A.-L., et al., *Comparison of two microalgal diets. 2. Influence on odorant composition and organoleptic qualities of raw oysters (Crassostrea gigas).* Journal of agricultural and food chemistry, 2003. **51**(7): p. 2011-2018.

22. Fu, R., et al., *A Study on the Formation and Change of Yesso Scallop Odor Characteristics by GC-O-MS, Sensory Evaluation, and Flavor-Precursor Analysis.* Aquaculture Research, 2023. **2023**.

23. Carrascon, V., et al., *Characterisation of the key odorants in a squid broth (Illex argentinus).* LWT - Food Science and Technology, 2014. **57**(2): p. 656-662.

24. Frank, D., et al., *Investigation of sensory and volatile characteristics of farmed and wild barramundi (Lates calcarifer) using gas chromatography− olfactometry mass spectrometry and descriptive sensory analysis.* Journal of Agricultural and Food Chemistry, 2009. **57**(21): p. 10302-10312.

25. Leduc, F., et al., *Evolution of volatile odorous compounds during the storage of European seabass (Dicentrarchus labrax).* Food Chemistry, 2012. **131**(4): p. 1304-1311.

26. Cai, L., et al., *The effect of magnetic nanoparticles plus microwave thawing on the volatile flavor characteristics of largemouth bass (Micropterus salmoides) fillets.* Food and Bioprocess Technology, 2019. **12**: p. 1340-1351.

27. Milo, C. and W. Grosch, *Detection of odor defects in boiled cod and trout by gas chromatography-olfactometry of headspace samples.* Journal of Agricultural and Food Chemistry, 1995. **43**(2): p. 459-462.

28. Milo, C. and W. Grosch, *Changes in the odorants of boiled salmon and cod as affected by the storage of the raw material.* Journal of Agricultural and Food Chemistry, 1996. **44**(8): p. 2366-2371.

29. Olafsdottir, G., et al., *Characterization of volatile compounds in chilled cod (Gadus morhua) fillets by gas chromatography and detection of quality indicators by an electronic nose.* Journal of Agricultural and Food Chemistry, 2005. **53**(26): p. 10140-10147.

30. Triqui, R., *Sensory and flavor profiles as a means of assessing freshness of hake (Merluccius merluccius) during ice storage.* European Food Research and Technology, 2006. **222**: p. 41-47.

31. Cayhan, G.G. and S. Selli, *Characterization of the key aroma compounds in cooked grey mullet (Mugil cephalus) by application of aroma extract dilution analysis.* Journal of agricultural and food chemistry, 2011. **59**(2): p. 654-659.

32. Salum, P., G. Guclu, and S. Selli, *Comparative evaluation of key aroma-active compounds in raw and cooked red mullet (Mullus barbatus) by aroma extract dilution analysis.* Journal of agricultural and food chemistry, 2017. **65**(38): p. 8402-8408.

33. Varlet, V., et al., *Comparison of odor-active volatile compounds of fresh and smoked salmon.* Journal of agricultural and food chemistry, 2006. **54**(9): p. 3391-3401.

34. Guo, H., et al., *Effects of electron‐beam irradiation on volatile flavor compounds of salmon fillets by the molecular sensory science technique.* Journal of Food Science, 2021. **86**(1): p. 184-193.

35. Triqui, R. and N. Bouchriti, *Freshness assessments of Moroccan sardine (Sardina pilchardus): comparison of overall sensory changes to instrumentally determined volatiles.* Journal of Agricultural and Food Chemistry, 2003. **51**(26): p. 7540-7546.

36. Prost, C., et al., *Effect of storage time on raw sardine (Sardina pilchardus) flavor and aroma quality.* Journal of Food Science, 2004. **69**(5): p. S198-S204.

37. Ganeko, N., et al., *Analysis of volatile flavor compounds of sardine (Sardinops melanostica) by solid phase microextraction.* Journal of Food Science, 2008. **73**(1): p. S83-S88.

38. Sérot, T., C. Regost, and J. Arzel, *Identification of odour‐active compounds in muscle of brown trout (Salmo trutta) as affected by dietary lipid sources.* Journal of the Science of Food and Agriculture, 2002. **82**(6): p. 636-643.

39. Selli, S., et al., *Characterization of aroma-active compounds in rainbow trout (Oncorhynchus mykiss) eliciting an off-odor.* Journal of Agricultural and Food Chemistry, 2006. **54**(25): p. 9496-9502.

40. Mahmoud, M.A.A. and A. Buettner, *Characterisation of aroma-active and off-odour compounds in German rainbow trout (Oncorhynchus mykiss). Part II: Case of fish meat and skin from earthen-ponds farming.* Food Chemistry, 2017. **232**: p. 841-849.

41. Cengiz, N., et al., *GC–MS-Olfactometric characterization of key odorants in rainbow trout by the application of aroma extract dilution analysis: Understanding locational and seasonal effects.* Food Chemistry, 2023. **407**: p. 135137.

42. Prost, C., T. Serot, and M. Demaimay, *Identification of the most potent odorants in wild and farmed cooked turbot (Scophtalamus maximus L.).* Journal of Agricultural and Food Chemistry, 1998. **46**(8): p. 3214-3219.

43. Sérot, T., et al., *Effect of dietary lipid sources on odour‐active compounds in muscle of turbot (Psetta maxima).* Journal of the Science of Food and Agriculture, 2001. **81**(14): p. 1339-1346.

44. Tanimoto, S., et al., *Qualitative changes in each part of yellowtail Seriola quinqueradiata flesh during cold storage.* Fisheries science, 2018. **84**: p. 135-148.

45. Kitabayashi, K., et al., *Effect of nitrogen gas packaging on odor development in yellowtail Seriola quinqueradiata muscle during ice storage.* Fisheries science, 2019. **85**: p. 247-257.

46. Hamakawa, Y., et al., *Effect of different heating conditions on odor of yellowtail Seriola quinqueradiata muscles.* Bioscience, Biotechnology, and Biochemistry, 2021. **85**(9): p. 2030-2041.
